# N-acetylcysteine attenuates oxidative-stress-associated apoptosis and collagen deposition after rat hindlimb ischemia-reperfusion injury

**DOI:** 10.1371/journal.pone.0357829

**Published:** 2026-09-10

**Authors:** Hideto Matsunaga, Ryuji Yonemitsu, Katsumasa Ideo, Junnosuke Ide, Masaki Shimada, Makoto Tateyama, Xiao Tian, Shu Takata, Kosei Takata, Shuntaro Tanimura, Yuto Shibata, Naoto Yoshimura, Kazuya Maeda, Junki Kawakami, Takahiro Arima, Yuki Kai, Soichiro Karata, Hikaru Goshogawa, Satoshi Hisanaga, Kazuki Sugimoto, Hironori Tanoue, Masaki Yugami, Takayuki Nakamura, Yusuke Uehara, Tetsuro Masuda, Takuya Tokunaga, Tatsuki Karasugi, Takeshi Miyamoto

**Affiliations:** Department of Orthopedic Surgery, Faculty of Life Sciences, Kumamoto University, Kumamoto, Japan; Universidade de Trás-os-Montes e Alto Douro: Universidade de Tras-os-Montes e Alto Douro, PORTUGAL

## Abstract

Proper use of fingers and limbs is crucial for people to carry out normal activities of daily living. Therefore, when fingers or limbs are severed accidentally, replantation is attempted whenever possible. However, even when replantation is successful and fingers or limbs are preserved, functional limitations often arise due to contractures in reattached fingers or limbs, although mechanisms underlying contractures or countermeasures to ameliorate them are not well understood. Here, using a rat femoral artery ischemia-reperfusion (I/R) model, we show that oxidative stress caused by accumulation of oxidative DNA damage occurs in gastrocnemius and soleus muscles after I/R induces muscle cell apoptosis. Moreover, we demonstrate that administration of the antioxidant N-acetyl cysteine (NAC) significantly suppresses oxidative stress accumulation and apoptosis induction in both muscles. We observed that collagen fibers accumulate in the gastrocnemius and soleus muscles after I/R in our model, and that collagen fiber accumulation was significantly suppressed by NAC administration. We demonstrate that adding hydrogen peroxide (H_2_O_2_), a reactive oxygen species (ROS), to an *in vitro* C2C12 myoblast culture system significantly increased expression of the apoptosis-inducing factors Bcl-2-associated X protein (BAX) and Caspase 3, while co-treatment with NAC significantly counteracted this increase and increased expression of the anti-apoptotic factor B-cell lymphoma 2 (Bcl2). Also using the C2C12 myoblast culture system, we show that H_2_O_2_ significantly increased expression of Cellular Communication Network Factor 2 (CCN2), which induces fibrosis, while co-addition of NAC with H_2_O_2_ significantly suppressed CCN2 induction. Our findings shed light on mechanisms underlying I/R injury in limbs and suggest countermeasures.

## Introduction

Amputation of fingers or limbs frequently occurs due to traumatic injuries such as traffic accidents or workplace accidents. Such losses often lead to significant functional impairment and cosmetic concerns, making replantation desirable. In the United States, it is reported that 45,000 cases of finger or limb amputations occur annually [1,2], with a replantation rate of approximately 14–18% performed for these injuries [3,4]. However, the survival rate of distal tissue following replantation surgery for amputated fingers or limbs is estimated to be between 48% and 97% [5–7], and this rate decreases as the time between amputation and restoration of blood flow to the distal tissue increases [5,8,9]. Furthermore, even when peripheral tissue survives via rapid restoration of interrupted blood flow, functional impairment, such as contractures of the fingers or limbs, remains a serious functional concern.

Ischemia-reperfusion (I/R) injury can also occur in various organs such as liver, brain, and heart [10–13]. Multiple factors, including adenosine triphosphate (ATP) depletion, ROS production, activation of innate immune inflammation, and release of Damage-Associated Molecular Patterns (DAMPs), are known to contribute to these types of injury in ischemic-reperfused tissues [10]. I/R injury varies by organ, exhibiting distinct characteristics, such as pulmonary edema in lungs [14]. In finger amputation, complete cessation of blood flow leads to various tissue injuries like necrosis and no-reflow phenomena following replantation, which reduce the survival rate of reattached fingers [5,15]. Various factors also reportedly participate in mechanisms underlying I/R injury in fingers and limbs [16–18]. To improve survival rate of reattached fingers, some have used cold storage of severed digits or limbs until replantation or administration of antioxidants and/or anti-inflammatory drugs after replantation surgery [19–23]. However, little is known about how functional impairment caused by contractures can be avoided to enhance successful reattachment of fingers.

Different animal models have been used to analyze I/R injury of various organs [24–28]. For models of finger or limb injury, the rat hind limb replantation model is often used, as vessel diameter is comparable to that of the human digital artery [29–31]. In this procedure, the limb is completely transected—including bone, muscles, and nerves—and then replanted via microvascular anastomosis of the femoral artery and vein. Another model involves occluding blood flow to the lower limb in animals using a tourniquet or clamping the femoral artery, followed by releasing the tourniquet or clamp to restore blood flow [32–34]. The former model has the advantage of reflecting the clinical reality of amputated fingers, but it also presents challenges: while results tend to vary due to complexity of the surgical procedure and all vascular sources are completely blocked during the ischemic phase and resulting in more severe I/R injury compared to simple occlusion models [31,35]. Lower limb tourniquet and femoral artery clamp models offer the advantages of simplicity and high reproducibility of results [36] but cannot completely block blood flow, resulting in a milder degree of I/R injury [37].

In the current study, we employed a model that induces peripheral I/R injury by first maximally obstructing peripheral blood flow using a combination of femoral artery clamping and a thigh tourniquet, followed by release of both. We found this technique simple and reproducible, and our analyses confirmed oxidative DNA damage and induction of terminal deoxynucleotidyl transferase dUTP nick end labeling (TUNEL)-positive apoptotic cells in gastrocnemius and soleus muscles after I/R. We show that administration of the antioxidant N-acetyl cysteine (NAC) significantly suppressed oxidative DNA damage and induction of TUNEL-positive apoptosis in both muscles. We also show induction of collagen fiber accumulation in gastrocnemius and soleus muscles after I/R, an activity thought to cause tissue contracture following I/R. However, these activities were also significantly suppressed by NAC administration. Taken together, herein we propose mechanisms for underlying oxidative-stress-associated skeletal muscle injury and collagen deposition after I/R in the limbs and suggest countermeasures.

## Materials and methods

### Animals and experimental design

Male Sprague–Dawley rats were purchased from Japan SLC, Inc. (Hamamatsu, Shizuoka, Japan). All rats were maintained in a pathogen-free facility under controlled environmental conditions with a 12:12-hour light:dark cycle at a stable temperature of 23 °C. The animals were provided with ad libitum access to water and a normal diet (ND) (CE-2; CLEA Japan Inc., Tokyo, Japan).

### NAC administration and hindlimb I/R model

Male Sprague–Dawley rats (8–10 weeks old; body weight, 291.6 ± 23.3 g) were allocated to either control (Ctrl) or N-acetyl-L-cysteine (NAC) group without a formal randomization procedure. There was no statistically significant difference in baseline body weight between the two groups (each with n = 4, exact P-values for Ctrl vs. NAC; P = 0.782 [1d], P = 0.998 [7d], P = 0.999 [14d]). To assess outcomes at different stages, independent cohorts of animals were sacrificed at each specific time point (cross-sectional design), with an exact sample size of n = 4 per group, per time point. Some animals were excluded from final analysis due to predefined technical criteria, such as surgical failure or unexpected death. A priori sample-size estimation (power analysis) was not performed due to difficulty in reliably estimating effect sizes and variances from previous studies or preliminary data. Instead, the sample size (n = 4 per group) was determined based on numbers commonly utilized in similar I/R models [38], strictly adhering to the ethical principle of minimizing animal use. Using an anesthesia box, rats were anesthetized by inhalation of isoflurane (4.0%, ~ 2.7 MAC for induction) and intraperitoneal administration of a triad of anesthetics (medetomidine hydrochloride 0.375 mg/kg + midazolam 2.0 mg/kg + butorphanol tartrate 2.5 mg/kg). After anesthetized rats were secured in a supine position, the hair on the right lower side was shaved and the skin moistened with alcohol. A longitudinal incision, approximately 1 cm in length, was made along the inner thigh, and the femoral arteries were dissected. To investigate its effects on I/R injury, NAC was administered intravenously (150 mg/kg) 15 minutes (min) before the initiation of reperfusion. Following this initial dose, the NAC group received continuous supplementation of NAC in drinking water (600 mg/L) throughout the experimental period [39], while the Ctrl group received an equivalent volume of saline intravenously 15 min before reperfusion and regular drinking water thereafter. For the I/R procedure, ischemia was induced in the right hindlimb by clamping the femoral artery for 4 hour (h), combined with applying a proximal rubber tourniquet to block collateral blood flow. Reperfusion was initiated by removal of both the clamp and tourniquet. The contralateral left hindlimb served as internal sham control, receiving only a skin incision and partial femoral artery exposure under the same anesthetic conditions.

### Animal ethics

Rats were euthanized using a humane procedure, taking into consideration alleviation of suffering. Using an anesthesia box, rats were anesthetized by inhalation of isoflurane and intraperitoneal administration of a triad of anesthetics (medetomidine hydrochloride 0.375 mg/kg + midazolam 2.0 mg/kg + butorphanol tartrate 2.5 mg/kg). After determining that pain relief was adequate, we euthanized rats by cervical dislocation. All animal experiments were carried out in accordance with the Institutional Guidelines on Animal Experimentation at Kumamoto University, and animal experiment procedures were approved by the Animal Studies Committee and the Institutional Animal Care and Use Committee at Kumamoto University, Japan. This study is reported in accordance with ARRIVE guidelines.

### Hindlimb blood flow assessment

Hindlimb blood flow was measured in separate cohorts at baseline (before surgery), 4 h after ischemia, and at 1, 7 and 14 days after reperfusion using a two-dimensional laser Doppler flowmetry system (OMEGA ZONE, Omega Wave Co., Tokyo, Japan). This laser speckle blood flow (LSBF) analyzing system was used to obtain representative images of perfused tissues. During the procedure, the same I/R protocol and NAC administration (intravenous injection, followed by supplying drinking water containing NAC) were performed as described above. To avoid data variations caused by environmental factors such as ambient light and temperature, hindlimb blood flow was expressed as the ratio of values obtained from the ischemic (right) versus contralateral control (left) hindlimb.

### Tissue preparation and sectioning

At indicated time points after reperfusion, rats were deeply anesthetized, and transcardial perfusion fixation was performed with 4% paraformaldehyde. The gastrocnemius (GAS) and soleus (SOL) muscles were then harvested and immersion-fixed in 4% paraformaldehyde for 24 hours at 4 °C. Tissues were then dehydrated through a graded series of ethanol, cleared in xylene, and embedded in paraffin. Paraffin-embedded tissues were then sectioned to 4 μm thickness using a microtome (REM-710; Yamato Kohki Industrial Co., Ltd., Saitama, Japan).

### Immunofluorescence staining

To evaluate cell apoptosis in muscle tissues, fluorescent immunohistochemistry was performed using the MEBSTAIN Apoptosis TUNEL Kit Direct (Medical & Biological Laboratories Co., Ltd., Nagoya, Japan) according to the manufacturer’s instructions. After deparaffinization and rehydration, tissue sections were subjected to antigen retrieval by incubation with Proteinase K (provided in the kit) for 30 min at 37 °C. Sections were blocked with 3% bovine serum albumin (BSA) in phosphate-buffered saline (PBS) for 1 h at room temperature to minimize non-specific binding. Sections were then stained with the TUNEL reaction mixture, and nuclei were counterstained with propidium iodide (PI; 29037−76; Nacalai Tesque, Inc., Kyoto, Japan). TUNEL-positive cells were detected and captured using a fluorescence microscope (BZ-X800, Keyence, Osaka, Japan). To quantify apoptotic cells, images were captured at 400 × magnification. Ten random fields from the GAS and five from the SOL were evaluated per section. The number of TUNEL-positive nuclei and the total number of PI-stained nuclei were counted in each field, and the apoptotic index was calculated as the percentage of TUNEL-positive nuclei relative to the total number of nuclei (TUNEL-positive nuclei/ total nuclei × 100).

### Immunohistochemistry

After deparaffinization and rehydration, sections were subjected to antigen retrieval by microwave treatment for 10 min in 10 mM citrate buffer solution (pH 6.0). Endogenous peroxidase activity was blocked with 3% H_2_O_2_ for 10 min. To minimize non-specific reactions, sections were incubated with Blocking One Histo (Nacalai Tesque, Inc., Kyoto, Japan) for 10 min at room temperature. Sections were then incubated overnight at 4°C with a mouse monoclonal antibody against 8-hydroxy-2’-deoxyguanosine (8-OHdG) (ab48508, 1:160; Abcam, Cambridge, UK). Samples were then incubated 1 h at room temperature with a horseradish peroxidase (HRP)-conjugated secondary antibody (ab97040, goat anti-mouse, 1:400; Abcam). Immunoreactivity was visualized using 3,3’-diaminobenzidine (DAB) chromogen, and nuclei were counterstained with hematoxylin. Sections were observed under a light microscope (BZ-X800, Keyence, Osaka, Japan). To quantify 8-OHdG-positive cells, images were captured at 400 × magnification. Ten random fields from the GAS and five from the SOL were evaluated per section. The number of 8-OHdG-positive nuclei and the total number of hematoxylin-stained nuclei were counted in each field, and the percentage of 8-OHdG-positive cells was calculated the number of 8-OHdG-positive nuclei relative to the total number of nuclei (8-OHdG-positive nuclei/ total nuclei × 100).

### Masson’s trichrome staining

Muscle fibrosis was evaluated by Masson’s trichrome staining using reagents from Wako Pure Chemical Industries (Osaka, Japan) and Muto Pure Chemicals (Tokyo, Japan). After deparaffinization and rehydration, paraffin sections were stained with Weigert’s iron hematoxylin (Wako) for 5 min, followed by staining with Ponceau xylidine-acid fuchsin solution (Wako) for 30 min. Sections were then incubated in a mixture of Aniline blue and Orange G (Muto) for 8 min. Between each staining step, sections were rinsed with 1% acetic acid. Collagen fibers were stained blue, nuclei black, and muscle fibers red. Sections were observed under a light microscope (BZ-X800, Keyence, Osaka, Japan). To evaluate collagen-positive areas quantitatively, images were captured at 400 × magnification. Ten random fields from the GAS and five from the SOL were evaluated per section. The percentage of collagen-positive area was calculated as the ratio of the area stained with aniline blue to the total tissue area (aniline blue-stained area/ total tissue area × 100).

### Histological assessment

Although investigators were not strictly blinded while conducting experimental procedures or image acquisition, observer bias was minimized during the subsequent histological quantification. To do so, we utilized automated image quantification software integrated into the BZ-X800 microscope system (Keyence, Osaka, Japan). Target areas and positive cells were mechanically detected and quantified across all samples based on uniform, pre-set threshold parameters.

### Cell culture

For *in vitro* experiments, C2C12 myoblast cells were cultured in growth medium (Dulbecco’s Modified Eagle Medium (DMEM)) supplemented with 10% fetal bovine serum (FBS), 50 U/mL penicillin, and 100 µg/mL streptomycin) and maintained at 37 °C in a humidified atmosphere with 5% carbon dioxide (CO_2_). Cells were treated with or without 4 μM H_2_O_2_ (Wako) for 4 h to induce oxidative stress. For some experiments, cells were pre-incubated with various concentrations of NAC (4, 20, 100, and 500 μM) for 15 min prior to H_2_O_2_ stimulation. For mitogen-activated protein kinase (MAPK) inhibitor analysis, cells were cultured in the presence of 4 μM H_2_O_2_ with 0.4 μM U0126 (MAPK/ERK (Extracellular signal-regulated kinase) 1/2 (MEK1/2) inhibitor) (S1460; Selleck Chemicals, Houston, TX, USA), 0.4 μM SB203580 (p38 MAPK inhibitor) (S1076; Selleck Chemicals), or 0.4 μM SP600125 (c-Jun N-terminal kinase (JNK) inhibitor) (#662005; Calbiochem, San Diego, CA, USA). An equivalent volume of PBS was used as a vehicle control.

### ROS assays

Intracellular ROS accumulation was evaluated using a Highly Sensitive 2’,7’-dichlorodihydrofluorescein diacetate (DCFH-DA)-ROS Assay Kit (Dojindo Molecular Technologies, Kumamoto, Japan) according to the manufacturer’s instructions. Briefly, after washing C2C12 cells twice with Hanks’ Balanced Salt Solution (HBSS), cells were incubated with DCFH-DA dye working solution for 30 min. Fluorescence signals were observed and captured using a fluorescence microscope (BZ-X800, Keyence, Osaka, Japan). Quantification of fluorescence intensity was performed using the BZ-X800 analyzer software (Keyence).

### Glutathione disulfide (GSSG)/ Glutathione (GSH) quantification

GSSG/GSH accumulation was evaluated using a GSSG/GSH Quantification Kit (G257; Dojindo Molecular Technologies, Kumamoto, Japan) according to the manufacturer’s instructions. Briefly, C2C12 cells (1 × 10^7^ cells) were washed with PBS and lysed with 10 mmol/L hydrogen chloride (HCl) through two freeze-thaw cycles. After adding 5% 5-sulfosalicylic acid (SSA) and centrifugation at 8,000 × g for 10 min, the supernatant was collected and diluted with deionized water to a final SSA concentration of 0.5%. Absorbance was then measured at 405 nm using a PowerScan HT microplate reader (DS Pharma Biomedical Co., Ltd., Osaka, Japan).

### Real-time polymerase chain reaction (PCR)

Total RNA was isolated from cells using TRIzol reagent (Invitrogen, Tokyo, Japan). Complementary DNA (cDNA) was synthesized from total RNA using PrimeScript RT Master Mix (Takara Bio Inc., Shiga, Japan). Real-time PCR was performed using TB Green Premix Ex Taq II (Takara Bio Inc.) on a Thermal Cycler Dice Real Time System (Takara Bio Inc.), according to the manufacturer’s instructions. Relative expression levels were determined using a standard curve generated from serially-diluted cDNA products. *Glyceraldehyde-3-phosphate dehydrogenase* (*Gapdh*) served as internal control. Mouse-specific primer sequences used for C2C12 cell analysis were as follows:

*Gapdh* forward: 5’-GTCATGGGTGTGAACCATGAGAAG-3’;
*Gapdh* reverse: 5’-AGTCTTCTGGGTGGCAGTGATG-3’;
*Bcl2* forward: 5’-CCTGTGGATGACTGAGTACCTG-3’;
*Bcl2* reverse: 5’-AGCCAGGAGAAATCAAACAGAGG-3’;
*Bax* forward: 5’-AGGATGCGTCCACCAAGAAGCT-3’;
*Bax* reverse: 5’-TCCGTGTCCACGTCAGCAATCA-3’;
*Caspase-3* (*Casp3*) forward: 5’-GGAGTCTGACTGGAAAGCCGAA-3’;
*Casp3* reverse: 5’-CTTCTGGCAAGCCATCTCCTCA-3’;
*Col1a1* forward: 5’-ACTGTCCCAACCCCCAAAG-3’;
*Collagen type 1 chain* (*Col1a1*) reverse: 5’-ACGTATTCTTCCGGGCAGAA-3’;
*Col3* forward: 5’-AACCTGGTTTCTTCTCACCCTTC-3’;
*Col3* reverse: 5’-ACTCATAGGACTGACCAAGGTGG-3’;
*Transforming growth factor beta 1* (*Tgfb1*) forward: 5’-CACCGGAGAGCCCTGGATA-3’;
*Tgfb1* reverse: 5’-TGTACAGCTGCCGCACACA-3’;
*Tgfb2* forward: 5’-CGAGGCGAGATTTGCAGGTATT-3’;
*Tgfb2* reverse: 5’-TTAGCAGGAGATGTGGGGTCTT-3’;
*Fibroblast growth factor 2* (*Fgf2*) forward: 5’-AAGCGGCTCTACTGCAAGAACG-3’;
*Fgf2* reverse: 5’-CCTTGATAGACACAACTCCTCTC-3’;
*Ccn2* forward: 5’-CAAAGCAGCTGCAAATACCA-3’;
*Ccn2* reverse: 5’-GGCCAAATGTGTCTTCCAGT-3

### Western blot analysis

C2C12 cells were serum-starved (no FBS) in DMEM for 24 h and then stimulated with 4 μM H_2_O_2_ for various times. Cell lysates were collected, and Western blot analysis was performed using polyclonal antibodies for ERK (#9102), phosphorylated ERK (#9106), p38 MAPK (#8690), phosphorylated p38 MAPK (#9211), JNK (#9102), and phosphorylated JNK (#9255). All antibodies were obtained from Cell Signaling Technology (Danvers, MA, USA) and used at a 1:1000 dilution.

### Statistical analysis

All numerical data are presented as means ± SD (standard deviation). The Shapiro-Wilk test was used to assess normality of data distribution before conducting statistical analyses. For histological comparisons between the ischemic (right) and contralateral sham (left) limbs in the same animal, a paired t-test was employed. For comparisons of the blood-flow ratio (ischemic relative to contralateral control limb) between treatment groups, we used Student’s t-test. For comparisons among three or more groups across different time points, muscles, and treatment groups, we performed one-way analysis of variance (ANOVA), followed by the Tukey–Kramer post hoc test to adequately control for multiple testing. A p-value less than 0.05 was considered statistically significant (*p < 0.05; **p < 0.01; NS, not significant, throughout the paper).

## Results

### Accumulation of oxidative DNA damage in rat gastrocnemius and soleus muscles is induced after I/R

**Fig 1:**
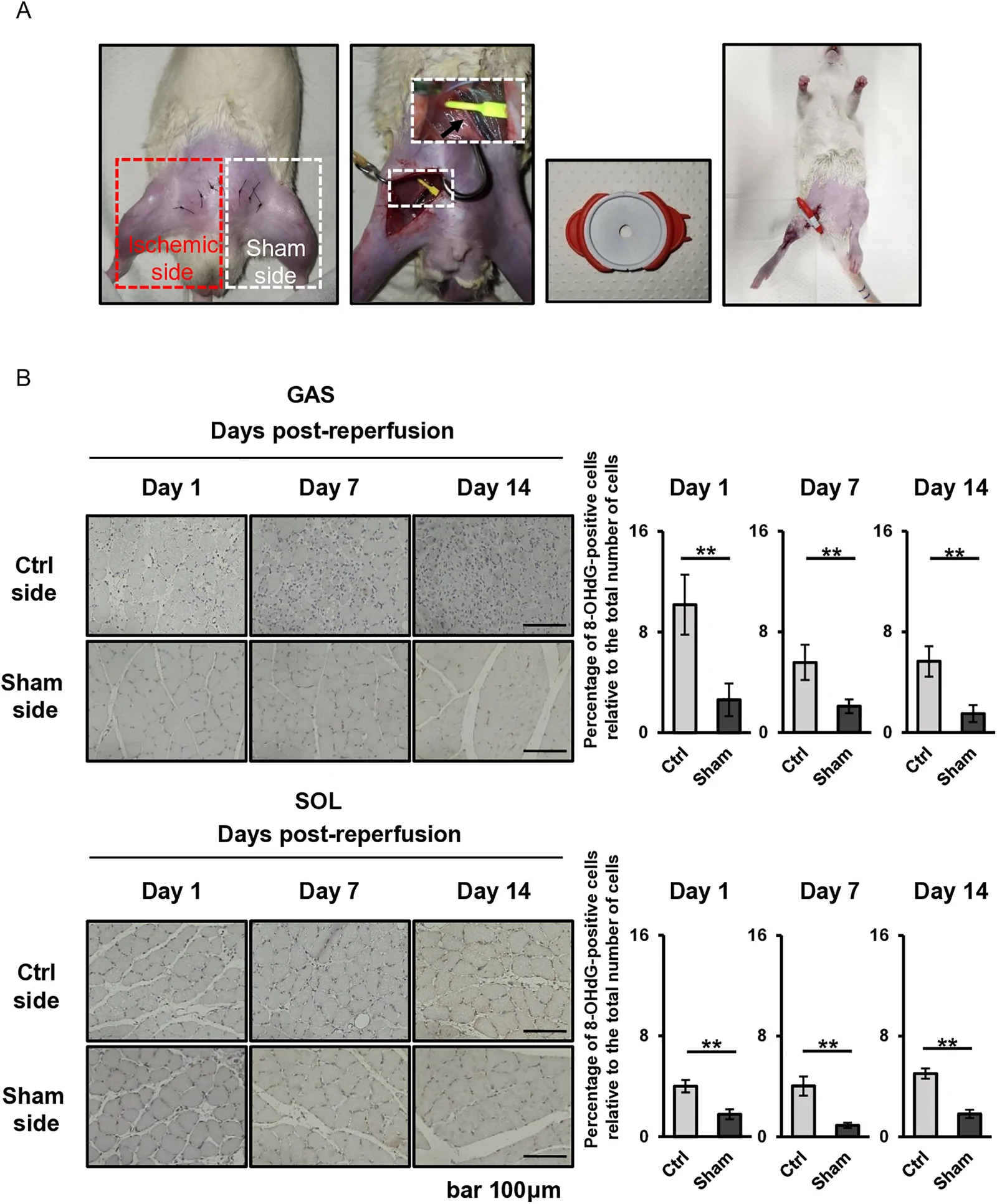
Oxidative stress is induced in rat hindlimb of an ischemia–reperfusion (I/R) model. (A) Representative photos illustrating the rat hindlimb I/R model. Ischemia was induced by clamping right femoral artery (black arrow) for 4 h, combined with application of a proximal rubber tourniquet to block collateral blood flow (leftmost and second-from-left panels). The left hindlimb, which served as a Sham control, received only a skin incision and partial femoral artery exposure. Tourniquet is shown in third panel from left. Far right shows a rat with the femoral artery clamped, the wound closed, and the tourniquet applied to the thigh. (B) (Left) Paraffin sections of GAS and SOL muscles prepared 1, 7 and 14 days after reperfusion (as shown in A) and stained with a biotin-conjugated anti-8-OHdG antibody. Immunoreactivity was visualized using the streptavidin-peroxidase method with 3,3’-diaminobenzidine (DAB) chromogen, and nuclei were counterstained with hematoxylin. Representative images are shown. Scale bar: 100 μm. Right, graphs show quantification of staining 8‑OHdG–positive cells shown as a proportion of total cells. Data are presented as mean ± SD (each with n = 4, *P < 0.05, **P < 0.01 [n.s. = not significant] vs. sham limb; all P < 0.01).

First, we established an animal model using rat hindlimbs as a limb I/R model by combining femoral artery clamping with a tourniquet applied to the thigh. As noted (see Introduction), although clinically relevant, such models tend to be inconsistent due to complexity of the surgical procedure [29,30]. In contrast, I/R injury is milder following application of vascular clamping or tourniquet methods, and these techniques offer a more stable and reproducible model [32–34,37]. Thus, here, we combined femoral artery clamping with a tourniquet applied to the thigh, releasing both after 4 hours to create an I/R model (Fig 1A). The contralateral lower limb underwent a sham procedure with a skin incision only. After reperfusion, tissue specimens were collected from the bilateral gastrocnemius and soleus muscles on days 1, 7, and 14. Tissue samples were then prepared and evaluated for oxidative DNA damaged using anti-8-OHdG antibody (Fig 1B). We confirmed significant accumulation of damaged DNA, based on 8-OHdG positivity, in both gastrocnemius and soleus muscles at all time points evaluated (Fig 1B).

### Administration of the antioxidant NAC significantly decreases oxidative DNA damage promoted by I/R in rat gastrocnemius and soleus muscle

**Fig 2:**
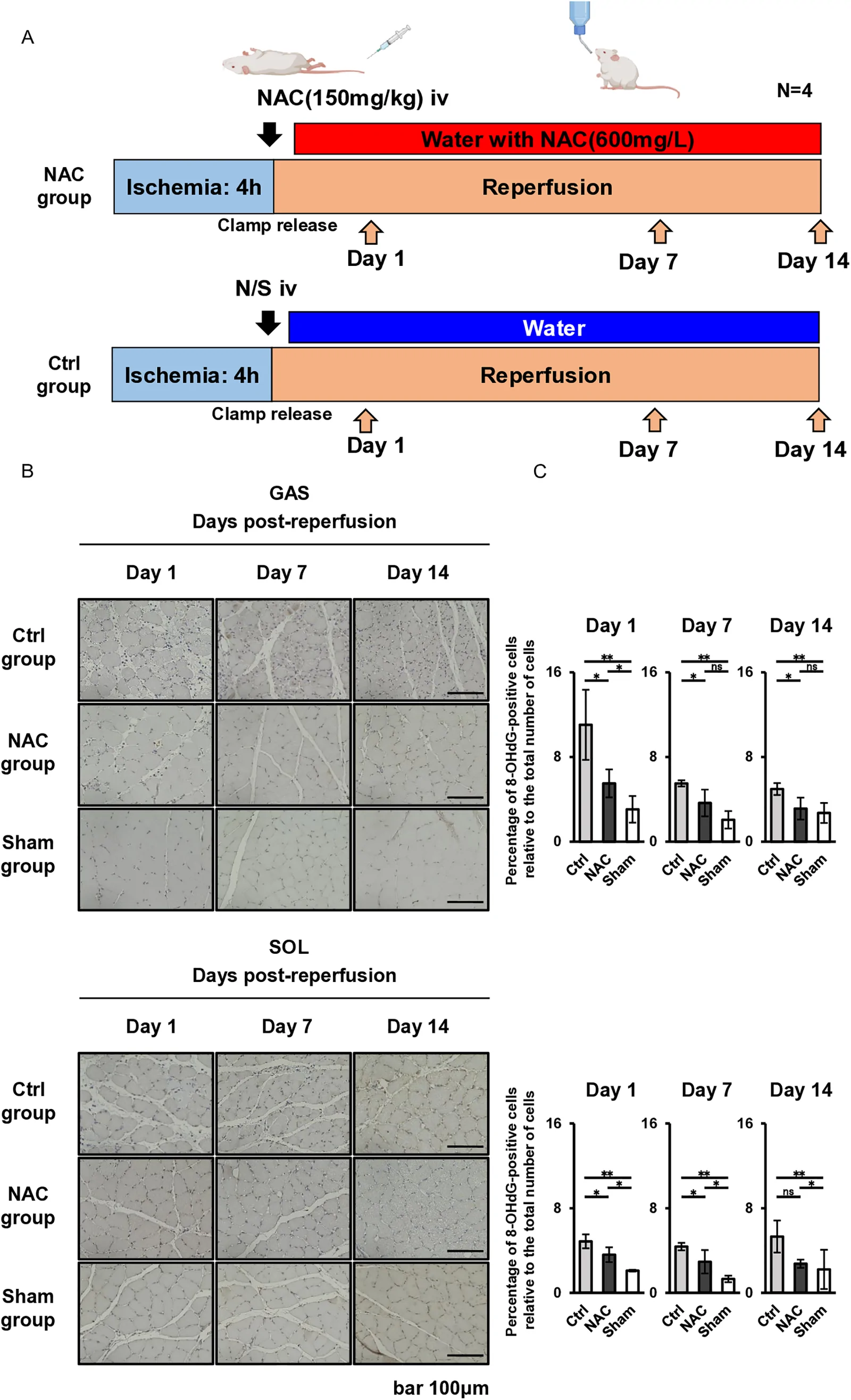
NAC administration reduces oxidative DNA damage in skeletal muscle in a rat limb I/R injury model. (A) Schematic showing N-acetyl-L-cysteine (NAC) administration protocol in rat hindlimb ischemia–reperfusion (I/R) model. The NAC group received NAC intravenously (150 mg/kg) 15 min before reperfusion, followed by continuous NAC supplementation (600 mg/L) in drinking water throughout the experimental period. The control (Ctrl) group was injected intravenously an equivalent volume of saline (N/S: Normal Saline) 15 min before reperfusion, and then NAC-free drinking water was provided thereafter. (B) Rats were allocated to Ctrl (saline) or NAC groups prior to I/R without a formal randomization procedure. Following reperfusion, animals were sacrificed on days 1, 7 and 14 and paraffin sections of GAS and SOL muscles were prepared. Sections were incubated with biotin-conjugated anti-8-OHdG antibody. Immunoreactivity was visualized using a streptavidin–peroxidase system with 3,3′-diaminobenzidine (DAB) chromogen. Nuclei were counterstained with hematoxylin. Representative images are shown. Scale bar: 100 μm. (C) Quantification of 8-OHdG-findings shown in (B). Shown is percentage of 8-OHdG-positive cells relative to total cells at each time point presented as mean ± SD (each with n = 4, *P < 0.05, **P < 0.01 [n.s. = not significant] for comparisons among sham limb, Ctrl, and NAC groups; exact P-values for Ctrl vs. NAC: GAS, P = 0.015 [1d], P = 0.039 [7d], P = 0.036 [14d]; SOL, P = 0.027 [1d], P = 0.041 [7d], P = 0.068 [14d]).

Next, we administered the antioxidant NAC to our rat I/R model (Fig 2A). After clamping the femoral artery and applying a thigh tourniquet for 4 hours, 150 mg/kg NAC (NAC group) or an equal volume of saline (Ctrl group) was administered intravenously to rats, and 15 minutes later, the clamp and tourniquet were released to restore blood flow. Thereafter, 600 mg/L NAC water (NAC group) or normal water (Ctrl group) was administered continuously in drinking water until the end of the experiment (Fig 2A). Relative to controls, NAC administration significantly suppressed accumulation of DNA damaged by oxidation in gastrocnemius muscles across all time points, and in soleus muscles on days 1 and 7, whereas this effect was not statistically significant on day 14 following administration (Fig 2B).

### NAC administration significantly suppresses muscle cell apoptosis induced by I/R in rat gastrocnemius and soleus

**Fig 3:**
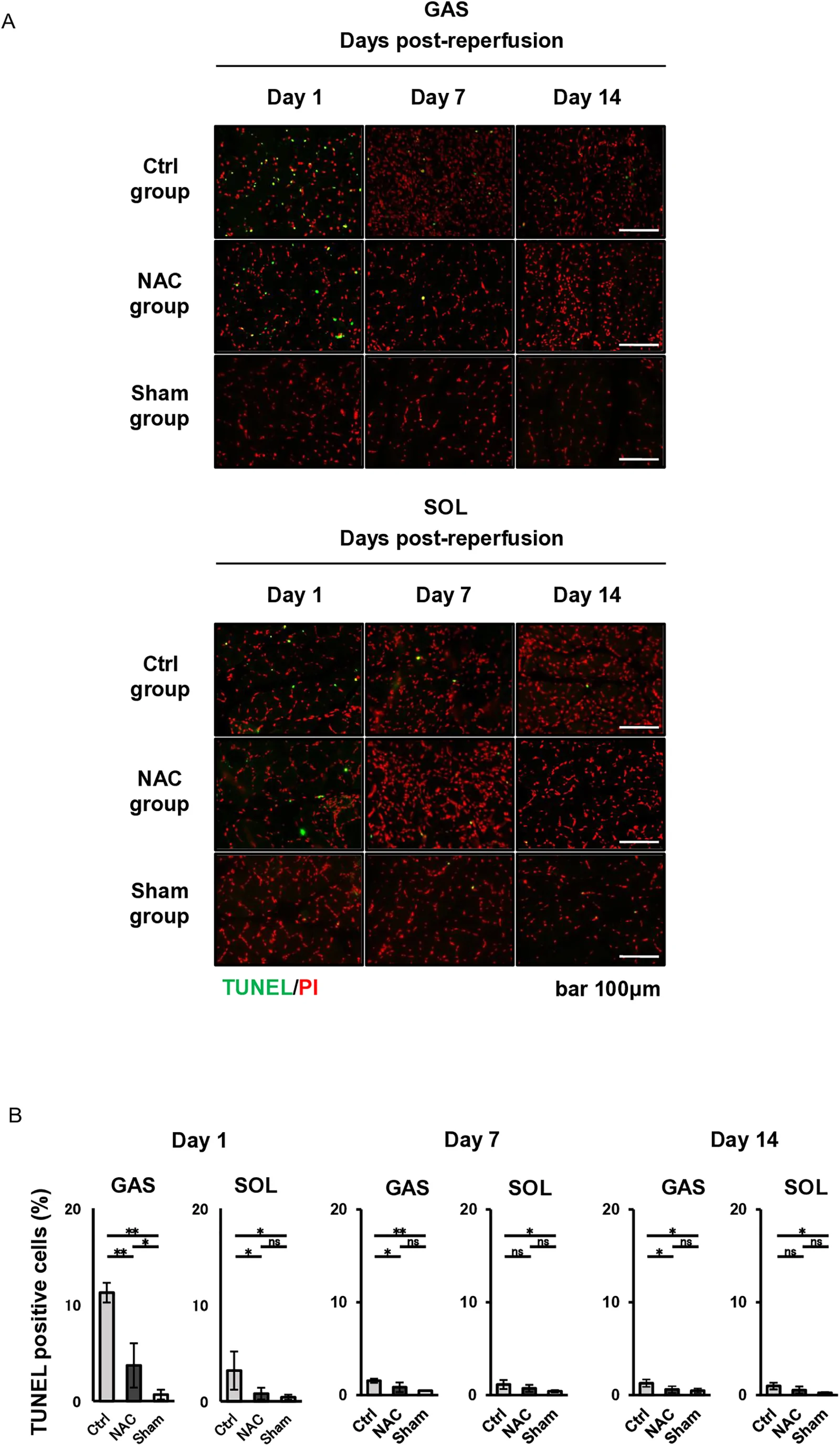
NAC administration blocks skeletal muscle cell apoptosis in a rat limb I/R injury model. (A) Prior to I/R, rats were assigned to Ctrl or NAC groups and maintained under conditions shown in Fig 2A. On days 1, 7 and 14 after reperfusion, paraffin sections of GAS and SOL muscles were prepared and labeled with biotin-dUTP using terminal deoxynucleotidyl transferase (TdT), followed by avidin-DTAF to identify apoptotic cells (TUNEL-positive, green). Nuclei were counterstained with propidium iodide (PI, red), and sections were observed under a fluorescence microscope. Scale bar: 100 μm. (B) Graphs show percentage of TUNEL-positive nuclei relative to total nuclei at each time point. Data are presented as mean percentage of TUNEL-positive nuclei relative to total nuclei ± SD (each with n = 4, *P < 0.05, **P < 0.01 [n.s. = not significant] for comparisons among sham limb, Ctrl, and NAC groups; exact P-values for Ctrl vs. NAC: GAS, P = 0.0001 [1d], P = 0.044 [7d], P = 0.046 [14d]; SOL, P = 0.049 [1d], P = 0.281 [7d], P = 0.171 [14d]).

To evaluate the extent of apoptotic cell death after potential I/R injury, we performed TUNEL assays of gastrocnemius and soleus tissues following treatment with or without NAC. In the Ctrl (no treatment) group, we observed significantly higher percentage of TUNEL-positive cells in both muscles at 1, 7 and 14 days after clamp and tourniquet removal compared with Sham group, and that staining was significantly suppressed by NAC treatment at all time points in gastrocnemius muscles, whereas in soleus muscles, significant suppression was observed only on day 1 (Fig 3A and 3B).

**Fig 4:**
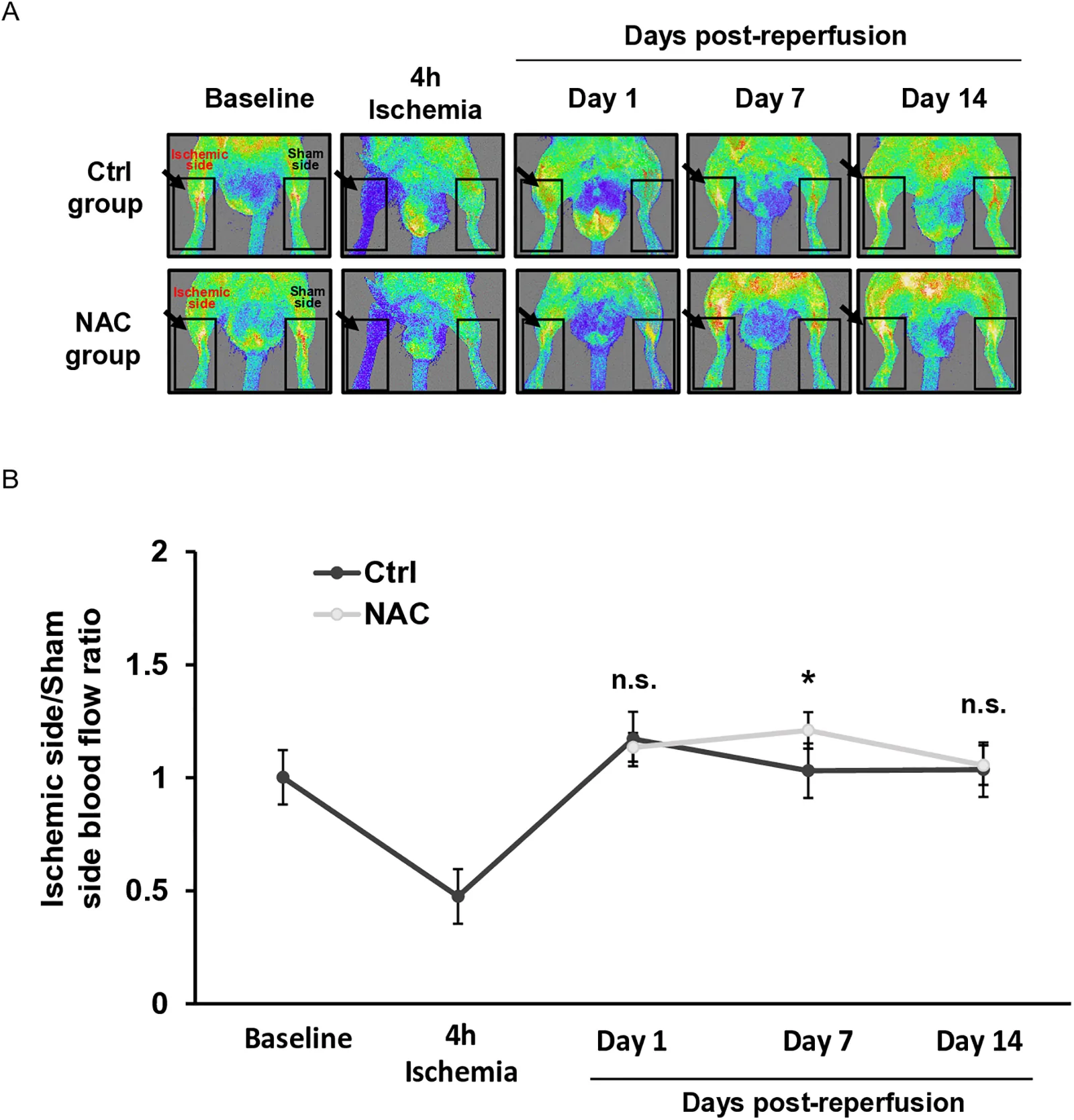
NAC administration increases blood flow to hindlimb in a rat limb I/R injury model. (A) Rats treated according to protocol shown in Fig 2A were subjected to two-dimensional laser Doppler flowmetry at each designated time point. Shown are representative images of hindlimb tissue at baseline, at 4 h after ischemia, and at indicated days after reperfusion. Black arrows indicate ischemic limb (right hindlimb). (B) Graph shows mean blood flow in ischemic relative to contralateral control limb. Data for both groups are presented as mean blood flow in ischemic versus control limb ± SD (each with n = 4, *P < 0.05, **P < 0.01 [n.s. = not significant] vs. Ctrl group; exact P-values for Ctrl vs. NAC: P = 0.48 [1d], P = 0.0153[7d], P = 0.69 [14d]).

We also assessed blood flow in both lower limbs of the NAC and Ctrl groups using two-dimensional laser Doppler flowmetry (Fig 4). In both groups, blood flow decreased to the same level 4 hours after femoral artery clamping and femoral tourniquet application, and recovered to baseline levels 1 day after clamp and tourniquet release (Fig 4A and 4B). Seven days after clamp and tourniquet release, blood flow was slightly but significantly higher in the NAC compared to the Ctrl group (Fig 4A and B), but by 14 days there were no differences between groups (Fig 4A and 4B). These findings suggest that amelioration of I/R injury promoted by NAC administration was likely due to decreases in oxidative stress. However, although the presence of NAC did not promote sustained differences in gross hindlimb blood-flow recovery, we cannot exclude the possibility that altered microvascular perfusion or vascular protection contributed to this effect.

### I/R induces tissue fibrosis, which in rat models is improved by NAC administration

**Fig 5:**
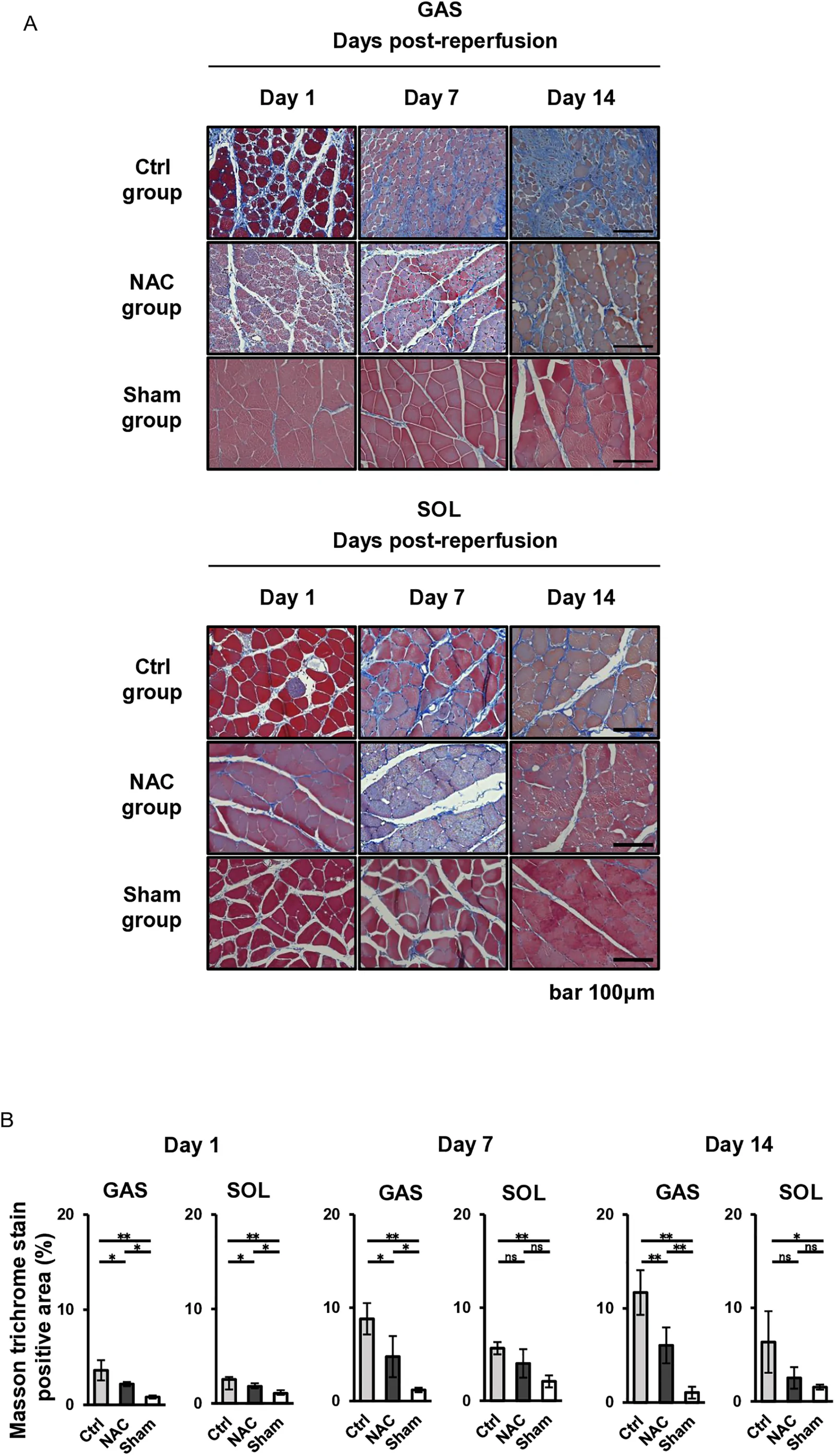
NAC administration suppresses skeletal muscle fibrosis in a rat limb I/R injury model. (A) Prior to I/R, rats were assigned to Ctrl or NAC groups and maintained as indicated in Fig 2A. On day 7 after reperfusion, paraffin sections of GAS and SOL muscles were prepared and stained using Masson’s trichrome protocol. Nuclei stained dark purple with iron hematoxylin; muscle fibers and cytoplasm stained red with acid fuchsin; and collagen fibers stained blue with aniline blue. Representative images are shown. Scale bar: 100 μm. (B) Quantification of collagen-positive (blue-stained) areas in GAS and SOL muscles. Graphs show proportion of the collagen-positive area relative to total tissue area. Data are presented as mean percentage of collagen-positive to total tissue areas ± SD (each with n = 4, *P < 0.05, **P < 0.01 [n.s. = not significant] for comparisons among sham limb, Ctrl, and NAC groups; exact P-values for Ctrl vs. NAC: GAS, P = 0.024 [1d], P = 0.015 [7d], P = 0.004 [14d]; SOL, P = 0.019 [1d], P = 0.117 [7d], P = 0.059 [14d]).

Even when replantation via vascular anastomosis is successful after finger or limb amputation, functional impairment due to tissue contracture often persists [40]. Since it is known that tissue contracture is induced by fibrosis [41], Masson trichrome staining was performed to evaluate tissue collagen deposition and fibrosis. In the control group (no treatment), after restoring blood flow following femoral artery clamping and tourniquet application, we observed significantly higher accumulation of Masson trichrome-stained collagen fibers in both GAS and SOL muscles on days 1, 7, and 14 days after reperfusion compared with Sham group, indicative of collagen deposition and fibrosis (Fig 5A and 5B). Interestingly, NAC administration (following protocol shown in Fig 2A) significantly reduced the I/R-induced Masson trichrome-stained positive areas in gastrocnemius muscles across all time points and in soleus muscles on day 1, but not on days 7 and 14 (Fig 5A and 5B).

### Oxidative stress induces expression of apoptosis-inducing factors in myoblasts

**Fig 6:**
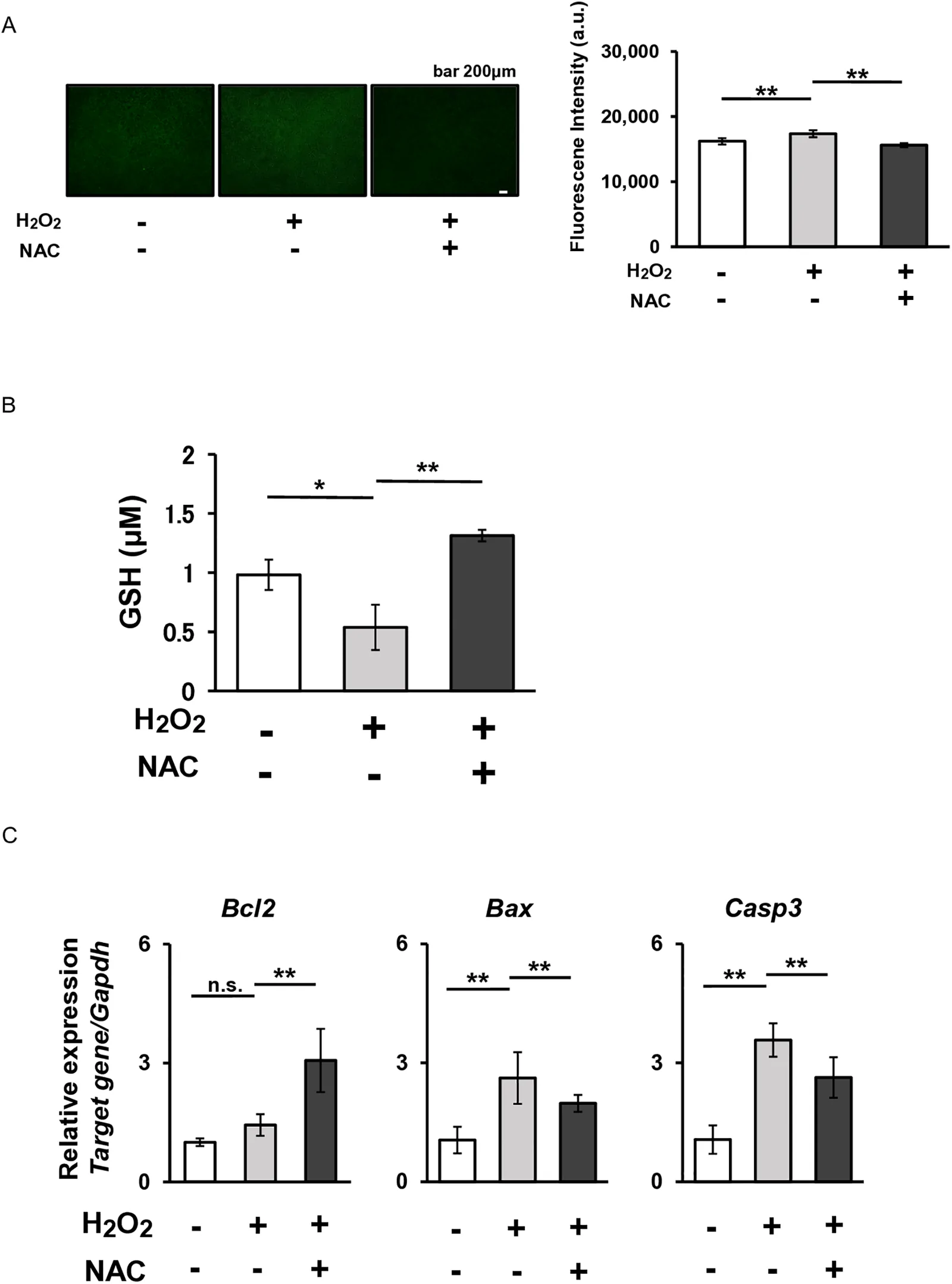
NAC suppresses H_2_O_2_-induced oxidative stress and expression of pro-apoptotic genes in C2C12 myoblasts. (A) C2C12 myoblasts were treated 4 h with or without H_2_O_2_ (4 μM) in the presence or absence of NAC (500 μM). (Left) ROS were visualized in representative images of indicated groups using the fluorescent probe DCFH-DA. (Right) Quantification of fluorescence intensity expressed in arbitrary units. Scale bar: 200 μm. (B) Quantification of GSH levels in indicated C2C12 myoblasts using a colorimetric assay based on redox-dependent chromogenic reactions. (C) Levels of *Bax*, *Casp3* or *Bcl2* mRNA in indicated C2C12 myoblasts relative to *Gapdh* based on qPCR. Data are presented as fold-change relative to the control cells not treated with H_2_O_2_ or NAC. For all analyses, data are shown as mean indicated gene expression relative to *Gapdh* ± SD (n = 6, *P < 0.05, **P < 0.01 vs. sham; n.s. = not significant).

To investigate mechanisms underlying tissue damage caused by oxidative stress induced by I/R, we conducted analysis in *in vitro* cultured C2C12 myoblasts, treated with and without H_2_O_2_, an oxidative stress agent (Fig 6). Treatment of C2C12 myoblasts for 4 hours with 4 μM H_2_O_2_ significantly increased ROS levels relative to untreated cells, based on by fluorescent staining with DCFH-DA. Co-treatment of cells with NAC significantly suppressed ROS levels induced by H_2_O_2_ (Fig 6A). Conversely, H_2_O_2_ treatment significantly decreased levels of the antioxidant glutathione (GSH) in C2C12 cells relative to controls, but those levels were significantly rescued by NAC co-administration (Fig 6B). H_2_O_2_ treatment also significantly increased expression of *Bax* and *Caspase 3* (*Casp3*), both apoptosis-inducing factors [42,43], an increase significantly blocked by NAC co-administration (Fig 6C). By contrast, expression of the apoptosis inhibitor *Bcl2* [44] in C2C12 myoblasts was unchanged by H_2_O_2_ stimulation, although its levels were significantly increased by NAC co-administration (Fig 6C).

### Oxidative stress induces expression of fibrosis-related factors in myoblasts

**Fig 7:**
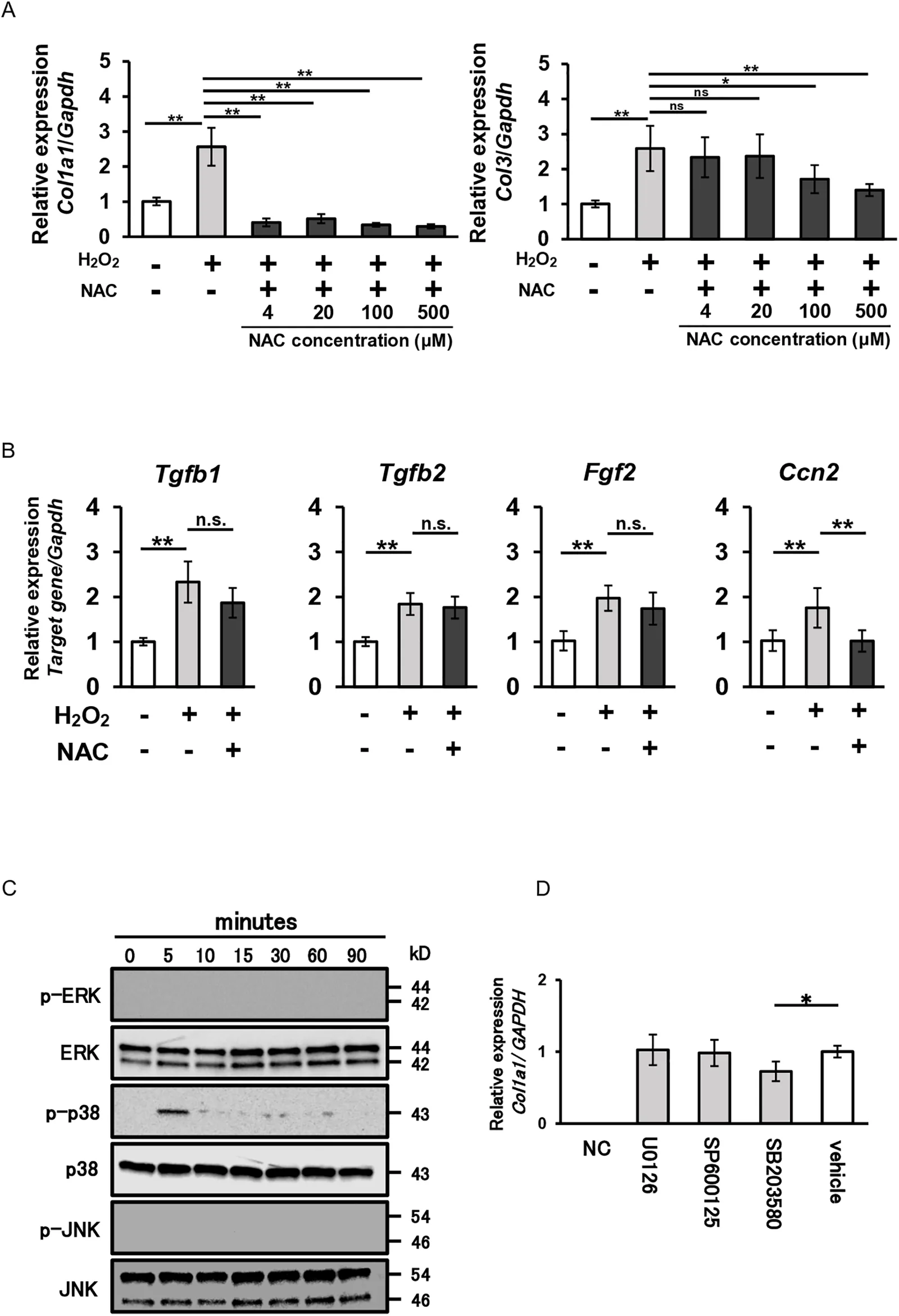
NAC inhibits H_2_O_2_-induced expression of genes associated with fibrosis and related signaling pathways in C2C12 myoblasts. (A) C2C12 myoblasts were treated 4 h with or without H_2_O_2_ (4 μM) in the presence or absence of NAC and assessed for levels *Col1a1* (left) or *Col3* (right) transcripts relative to *Gapdh* based on qPCR. Data are presented as fold-change relative to control cells not treated with either H_2_O_2_ or NAC. Data are shown as mean ± SD (n = 6, *P < 0.05, **P < 0.01 vs. sham; n.s. = not significant). (B) C2C12 myoblasts were treated as described in (A) and then assessed for transcript levels of *Tgfb1*, *Tgfb2*, *Fgf2* or *Ccn2* relative to *Gapdh,* based on qPCR. Data are presented as fold-change relative to control cells not treated with H_2_O_2_ or NAC. Data are shown as mean ± SD (n = 6, *P < 0.05, **P < 0.01 vs. sham; n.s. = not significant). (C) C2C12 cells were serum-starved for 24 h and stimulated with 4 μM H_2_O_2_ for indicated times. Then, cell lysates were collected, and phosphorylated and non-phosphorylated forms of ERK, p38 MAPK and JNK protein were examined by western blot. Representative images are shown. (D) C2C12 cells were cultured with 4 μM H_2_O_2_ in the presence or absence (vehicle) of a MEK1/2 inhibitor (0.4 μM U0126), a p38 MAPK inhibitor (0.4 μM SB203580), or a JNK inhibitor (0.4 μM SP600125) for 4 h, and *Col1a1,* and *Gapdh* expression was analyzed by qPCR. NC, no template control. (each with n = 6, *P < 0.05, **P < 0.01 [n.s. = not significant] for comparisons among vehicle, U0126, SB203580, and SP600125 groups; exact P-values for vehicle vs. SB203580: P = 0.036).

We next analyzed effects of oxidative stress on fibrosis in C2C12 cells (Fig 7). Treating C2C12 myoblasts 4 hours with 4 μM H_2_O_2_ significantly increased expression of collagen genes *Col1a1* and *Col3* (Fig 7A), an effect significantly suppressed by NAC co-administration (Fig 7A). The expression of *Tgfb1*, *Tgfb2*, *Fgf2*, and *Ccn2*, all factors associated with tissue fibrosis [45–47], significantly increased in C2C12 myoblasts following H_2_O_2_ treatment (Fig 7B), and among these factors, increased *Ccn2* expression was significantly suppressed when NAC was co-administered together (Fig 7B).

Finally, we asked which signaling pathways are activated by oxidative stress to induce fibrosis in myoblasts. To do so, we treated C2C12 myoblasts after 24-hour serum-starvation with 4 μM H_2_O_2_ for 90 minutes and collected cell lysates at time zero and various time points thereafter for Western blot analysis (Fig 7C). For that analysis we used antibodies specific for total and phosphorylated forms of proteins in the MAPK pathway, namely p38, JNK, and ERK. Interesting, we observed that H_2_O_2_ treatment specifically increased p38 phosphorylation in C2C12 myoblasts (Fig 7C). We then cultured C2C12 myoblasts with or without H_2_O_2_ in the presence or absence (vehicle) of p38 (SB203580), JNK (SP600125) or ERK (U0126) inhibitor, and we found that H_2_O_2_-induced *Col1a1* expression was specifically and significantly inhibited by p38 inhibitor (Fig 7D).

## Discussion

I/R injury resulting in functional impairment can occur in various tissues and organs [10–13], including during surgical reattachment of amputated fingers or limbs. Currently, better approaches to restore sufficient functionality after surgical limb or finger reattachment are needed [40]. Complications following replantation surgery for finger amputation include not only loss of that finger due to necrosis, but sensory disturbances, numbness, limited joint range of motion and contractures in the reattached finger. In this study, we employed a rat femoral artery clamp and femoral tourniquet I/R injury model and demonstrated that oxidative stress induced in distal tissues promotes apoptosis of muscle cells and fibrosis in muscle. We found that these impairments were significantly inhibited by administration of the antioxidant agent NAC, although the magnitude and statistical significance of the effect varied by muscle type and time point.

Replantation of severed fingers or limbs requires surgical reconstruction of the entire tissue complex and involves not only arterial and venous reanastomosis, but also bone fixation, and tendon, ligament and nerve suturing, often leading to a constellation of postoperative complications [48–51]. To develop countermeasures, it is necessary to define mechanisms underlying subsequent impairments.

Early postoperative complications include peripheral blood flow impairment and coldness due to arterial anastomotic failure, thrombosis, or vasospasm [9]. In our model, improvement in blood flow was promptly confirmed using laser Doppler flowmetry after blood flow was restored (Fig 3A). Venous stasis is also a significant complication following digital reattachment surgery; when it occurs, treatments such as phlebotomy or medical leech therapy may be employed [52,53]. By contrast, in our model, the veins remain intact. However, oxidative stress accumulation and muscle cell apoptosis occur as early as one day after reperfusion, even when arterial and venous blood appears fairly normal, suggesting the need for intervention during reperfusion surgery. Indeed, we show that intraoperative and postoperative NAC administration significantly suppresses ROS accumulation and induction of apoptosis following I/R.

Late complications of finger replantation surgery include contractures and restricted range of motion in the affected finger [40,48,54], all of which impair activities of daily living (ADL) and decrease patient satisfaction, necessitating intervention. Clinically, it was considered that contractures resulted from external fixation required after bone fixation or suturing of vessels and tendons, or from tissue adhesion during external fixation. In our animal model, bone fixation and soft tissue suturing were not performed; therefore, the affected limb was not immobilized after I/R. Moreover, an important limitation of this study is that we did not assess contractures or functional impairment in our animal models. Nevertheless, collagen deposition appeared one day after I/R. Such I/R-induced fibrosis was significantly improved by NAC administration immediately after surgery, suggesting it arises from oxidative stress. Meanwhile, it is known that NAC has wide-ranging effects beyond ROS scavenging, such as regulating intracellular thiol redox balance, glutathione metabolism, inflammatory signaling, mitochondrial stress, and endothelial function [55,56]. Indeed, here, we demonstrated that NAC suppresses oxidative DNA damage in an *in vivo* I/R model and that it suppresses ROS in an *in vitro* C2C12 myoblast model; however, it is also possible that NAC mitigates tissue damage following I/R through multiple biological pathways.

ROS exert biological effects by activating various signaling pathways, among them, all three MAPK pathways, namely, p38, JNK and ERK [57]. These three pathways are functionally diverse: ERK primarily mediates cell survival and proliferation, p38 regulates inflammatory and fibrotic responses, and JNK governs stress-induced apoptosis. In chondrocytes, ROS reportedly induce chondrocyte hypertrophy via p38 activation [58]. Here, using C2C12 myoblasts as a model, we observed p38 pathway activation in parallel with ROS induction in H_2_O_2_-treated cells. We also observed that ROS-induced *Col1a1* upregulation in those cells was significantly inhibited by treating cells with a p38 inhibitor. H_2_O_2_-induced *Ccn2*, *Col3*, *Tgfb1,* or *Tgfb2* expression was also slightly suppressed by p38 inhibition (S1 Fig), although that decrease was not statistically significant. These findings suggest that fibrotic phenotypes promoted by oxidative stress seen in our model may be due, at least in part, to p38 pathway activation. Although tissue fibrosis *in vivo* after I/R is induced by various cell types [59], including myoblasts, our *in vitro* C2C12 myoblast model suggests that myoblasts may contribute to profibrotic signaling but may not account fully for collagen accumulation *in vivo*.

NAC exerts antioxidant effects by increasing glutathione levels [60]. We confirmed a that GSH levels were significantly increased in cultured C2C12 myoblasts after NAC administration. In animal studies, NAC treatment reportedly significantly counteracts dysfunction and impaired cell proliferation in hematopoietic stem cells and proliferating chondrocytes caused by ROS accumulation [58,61]. Our findings may suggest that NAC administration can prevent tissue damage following replantation of severed fingers. Clinically, NAC is approved as an expectorant and antidote for acetaminophen poisoning [62,63] and is sold as a dietary supplement in many countries. While excessive intake may cause gastrointestinal symptoms such as nausea and vomiting, its safety for human use is well established. These and our findings support further investigation of NAC as a potential adjunctive therapy to attenuate oxidative-stress-associated skeletal muscle injury after I/R. However, future studies using functional, biomechanical, and clinically relevant replantation models, and functional endpoints are required to determine whether this strategy can prevent contracture or enhance functional recovery after replantation.

A limitation of our current model is that it clamps and then releases the femoral artery with tourniquet; thus, limb amputation and subsequent reattachment as seen in clinical settings are not performed. While our method is simple and yields highly reproducible results, the limb amputation–reattachment animal model involves complex procedures, including not only arterial and venous anastomosis, tendon and muscle suturing, and bone fixation. The complexity of these procedures makes it difficult to achieve reproducible results and may complicate tissue damage and recovery. Also, contracture following reattachment of a severed finger is caused not only by fibrosis resulting from I/R in various cells, including myoblasts, but also by scar formation due to tissue damage and immobilization after reattachment. These factors must be fully recognized when considering application of our study to the reattachment of amputated fingers.

Taken together, we demonstrate a strategy to prevent muscle cell apoptosis and tissue fibrosis by inhibiting ROS and potentially blocking aberrant MAPK signaling in limb I/R injury. We believe these findings could enhance attenuation of oxidative-stress-associated skeletal muscle injury and collagen deposition in patients after I/R.

## Supporting information

S1 Fig Effect of p38 MAPK inhibition on expression of various profibrotic genes in H_2_O_2_-stimulated C2C12 cells. C2C12 cells were cultured with 4 μM H_2_O_2_ in the presence or absence (vehicle) of a MEK1/2 inhibitor (0.4 μM U0126), a p38 MAPK inhibitor (0.4 μM SB203580), or a JNK inhibitor (0.4μM SP600125) for 4 h. Levels of *Ccn2*, *Col3a1*, *Tgfb1*, and *Tgfb2* transcripts were then analyzed by qPCR. NC, no template control (each with n = 6, *P < 0.05, **P < 0.01 [n.s. = not significant] for comparisons among vehicle, U0126, SB203580, and SP600125 groups; exact P-values for vehicle vs. SB203580: P = 0.107 [*Ccn2*], P = 0.702 [*Col3*], P = 0.821 [*Tgfb1*], P = 0.999 [*Tgfb2*]).
(PDF)

S1 Dataset This file contains the raw numerical data for all quantitative analyses presented in the study, including individual values for each experiment. (PDF)
