# Supplementary material for: N-acetylcysteine attenuates oxidative-stress-associated apoptosis and collagen deposition after rat hindlimb ischemia-reperfusion injury
Source: PLoS One. 2026 Sep 10;21(9):e0357829. doi: 10.1371/journal.pone.0357829 (PMC13561352; doi:10.1371/journal.pone.0357829)
Supplement: Supplementary file 1 [file pone.0357829.s001.pdf]

S1 Fig. Effect of p38 MAPK inhibition on expression of various profibrotic genes in H_2O_2-stimulated C2C12 cells.

C2C12 cells were cultured with 4 µM H_2O_2 in the presence or absence (vehicle) of a MEK1/2 inhibitor (0.4 µM U0126), a p38 MAPK inhibitor (0.4 µM SB203580), or a JNK inhibitor (0.4µM SP600125) for 4 h. Levels of *Ccn2*, *Col3a1*, *Tgfb1*, and *Tgfb2* transcripts were then analyzed by qPCR. NC, no template control (each with n = 6, *P < 0.05, **P < 0.01 [n.s. = not significant] for comparisons among vehicle, U0126, SB203580, and SP600125 groups; exact P-values for vehicle vs. SB203580: P = 0.107 [*Ccn2*], P = 0.702 [*Col3*], P = 0.821 [*Tgfb1*], P = 0.999 [*Tgfb2*])

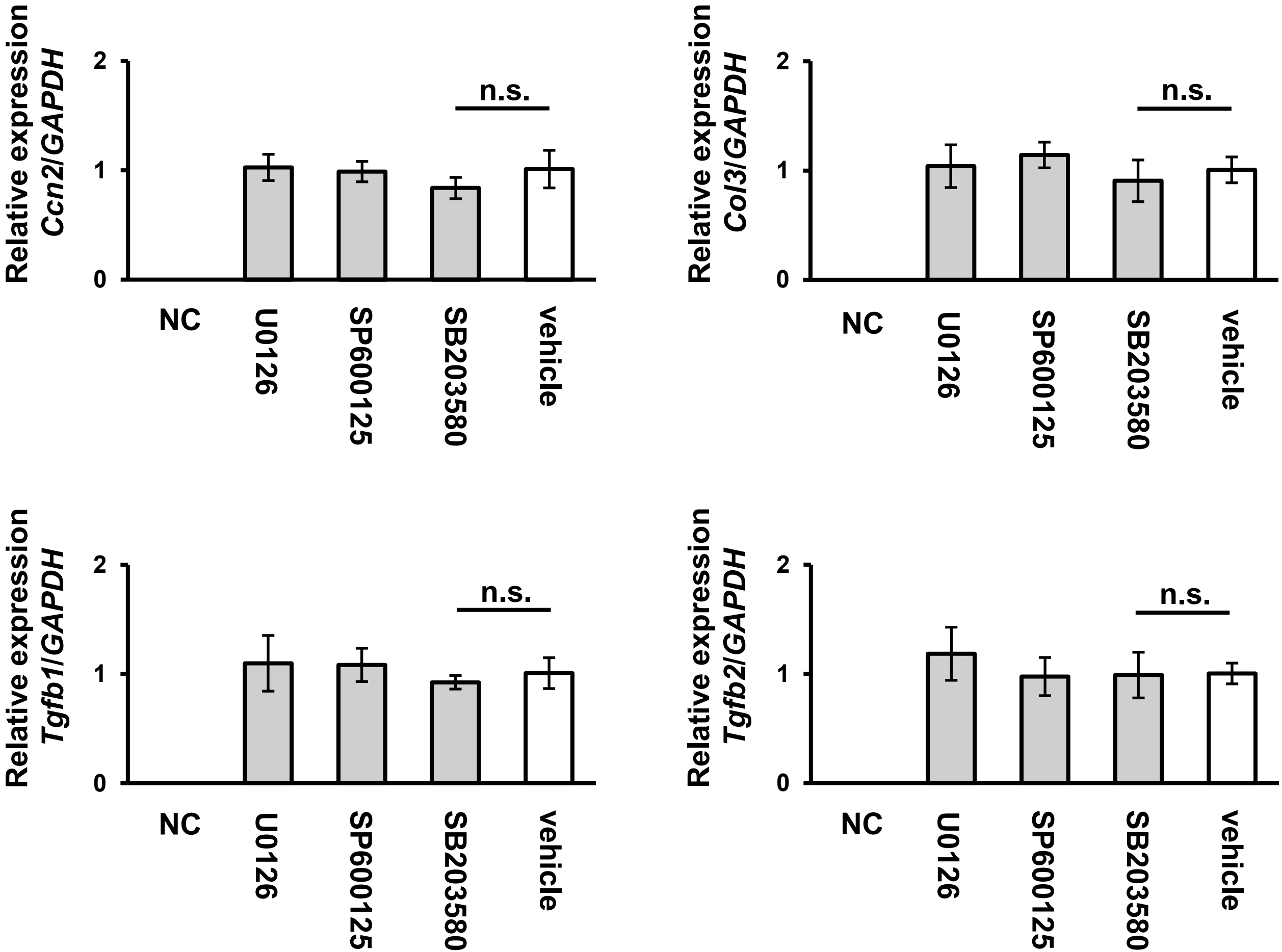
Relative expression
Ccn2/GAPDH
2
1
0
n.s.
NC
U0126
SP600125
SB203580
vehicle
Relative expression
Col3/GAPDH
2
1
0
n.s.
NC
U0126
SP600125
SB203580
vehicle
Relative expression
Tgfb1/GAPDH
2
1
0
n.s.
NC
U0126
SP600125
SB203580
vehicle
Relative expression
Tgfb2/GAPDH
2
1
0
n.s.
NC
U0126
SP600125
SB203580
vehicle
