# Supplementary material for: N-acetylcysteine attenuates oxidative-stress-associated apoptosis and collagen deposition after rat hindlimb ischemia-reperfusion injury
Source: PLoS One. 2026 Sep 10;21(9):e0357829. doi: 10.1371/journal.pone.0357829 (PMC13561352; doi:10.1371/journal.pone.0357829)
Supplement: Supplementary file 2 [file pone.0357829.s002.pdf]

Body weight

	Figure 1		
Sample	Day 1	Day 7	Day 14
1	294.4	327	270.4
2	289.7	306.4	280.5
3	278.5	280	276.5
4	282.5	297	308.2
Average	286.28	302.60	283.90
SD	7.1285	19.5952	16.7238

	Figure 2-5					
	Day 1		Day 7		Day 14	
Sample	Ctrl group	NAC group	Ctrl group	NAC group	Ctrl group	NAC group
1	365.5	315.8	292.5	260.4	323.6	295.5
2	288.5	290.5	301.3	288.4	298.9	292.4
3	294.8	275.5	258	272	297.3	290.4
4	323.6	285.6	247.4	325.5	256	266.7
Average	318.1	291.85	274.8	286.58	293.95	286.25
SD	35.1001	17.1446	26.1313	28.3787	28.0181	13.2011

Figure 1.

GAS

Day1						
	Ctrl side			Sham side		
Sample	8-OHdG-positive cells	Total counted cells	%	8-OHdG-positive cells	Total counted cells	%
1	85	674	12.6113	32	828	3.8647
2	100	869	11.5075	28	817	3.4272
3	61	842	7.2447	16	738	2.168
4	64	687	9.3159	7	699	1.0014
Average	77.5	768	10.1698	20.75	770.5	2.6153
SD	18.4120	101.7743	2.3830	11.4127	62.2816	1.2942

Day7						
	Ctrl side			Sham side		
Sample	8-OHdG-positive cells	Total counted cells	%	8-OHdG-positive cells	Total counted cells	%
1	192	3236	5.9333	23	867	2.6528
2	137	1856	7.3815	17	697	2.4390
3	96	2257	4.2534	11	718	1.5320
4	122	2581	4.7269	12	697	1.7217
Average	136.75	2482.5	5.5738	15.75	744.75	2.0864
SD	40.5411	583.3287	1.3973	5.5000	82.0990	0.5433

Day14						
	Ctrl side			Sham side		
Sample	8-OHdG-positive cells	Total counted cells	%	8-OHdG-positive cells	Total counted cells	%
1	221	3645	6.0631	12	810	1.4815
2	111	1989	5.5807	15	687	2.1834
3	243	3515	6.9132	12	679	1.7673
4	45	1115	4.0359	3	513	0.5848
Average	155	2566	5.6482	10.5	672.25	1.5042
SD	93.3381	1225.1764	1.2078	5.1962	121.9272	0.6773

SOL

Day1						
	Ctrl side			Sham side		
Sample	8-OHdG-positive cells	Total counted cells	%	8-OHdG-positive cells	Total counted cells	%
1	46	890	5.1685	16	736	2.1739
2	38	1008	3.7698	12	609	1.9704
3	27	677	3.9882	8	527	1.5180
4	25	815	3.0675	8	566	1.4134
Average	34	847.5	3.9985	11	609.5	1.7690
SD	9.8319	138.6759	0.8733	3.8297	90.7396	0.3624

Day7						
	Ctrl side			Sham side		
Sample	8-OHdG-positive cells	Total counted cells	%	8-OHdG-positive cells	Total counted cells	%
1	58	1389	4.1757	6	599	1.0017
2	22	666	3.3033	6	591	1.0152
3	44	1216	3.6184	6	589	1.0187
4	39	774	5.0388	4	671	0.5961
Average	40.75	1011.25	4.0340	5.5	612.5	0.9079
SD	14.8633	346.4596	0.7608	1.0000	39.2386	0.2080

Day14						
	Ctrl side			Sham side		
Sample	8-OHdG-positive cells	Total counted cells	%	8-OHdG-positive cells	Total counted cells	%
1	65	1367	4.7549	7	496	1.4113
2	35	723	4.8409	7	416	1.6827
3	59	1048	5.6298	10	459	2.1786
4	36	742	4.8518	8	407	1.9656
Average	48.75	970	5.0194	8	444.5	1.8096
SD	15.5000	303.6917	0.4092	1.4142	41.1542	0.3343

Figure 2.

GAS

Day1									
	Ctrl group			NAC group			Sham group		
Sample	8-OHdG-positive cells	Total counted cells	%	8-OHdG-positive cells	Total counted cells	%	8-OHdG-positive cells	Total counted cells	%
1	249	1701	14.6384	60	805	7.4534	42	860	4.8837
2	119	978	12.1677	33	733	4.5020	9	319	2.8213
3	59	555	10.6306	44	884	4.9774	10	414	2.4155
4	26	386	6.7358	46	900	5.1111	8	382	2.0942
Average	113.25	905	11.0431	45.75	830.5	5.5110	17.25	493.75	3.0537
SD	98.3476	586.1757	3.3123	11.0868	77.1341	1.3211	16.5202	247.3356	1.2558

Day7									
	Ctrl group			NAC group			Sham group		
Sample	8-OHdG-positive cells	Total counted cells	%	8-OHdG-positive cells	Total counted cells	%	8-OHdG-positive cells	Total counted cells	%
1	109	2140	5.0935	102	1897	5.3769	10	828	1.2077
2	144	2536	5.6782	21	650	3.2308	7	420	1.6667
3	122	2124	5.7439	52	1426	3.6466	11	353	3.1161
4	55	993	5.5388	69	2893	2.3851	14	606	2.3102
Average	107.5	1948.25	5.5136	61	1716.5	3.6598	10.5	551.75	2.0752
SD	37.8638	664.7327	0.2929	33.7935	937.8246	1.2593	2.8868	213.0076	0.8283

Day14									
	Ctrl group			NAC group			Sham group		
Sample	8-OHdG-positive cells	Total counted cells	%	8-OHdG-positive cells	Total counted cells	%	8-OHdG-positive cells	Total counted cells	%
1	118	2830	4.1696	31	1105	2.8054	14	603	2.3217
2	68	1340	5.0746	26	1193	2.1794	14	560	2.5000
3	117	2139	5.4698	23	784	2.9337	11	564	1.9504
4	78	1503	5.1896	43	937	4.5891	18	439	4.1002
Average	95.25	1953	4.9759	30.75	1004.75	3.1269	14.25	541.5	2.7181
SD	26.0176	678.7228	0.5626	8.8081	181.4835	1.0290	2.8723	71.0329	0.9495

SOL

Day1									
	Ctrl group			NAC group			Sham group		
Sample	8-OHdG-positive cells	Total counted cells	%	8-OHdG-positive cells	Total counted cells	%	8-OHdG-positive cells	Total counted cells	%
1	46	1116	4.1219	33	781	4.2254	18	914	1.9694
2	44	806	5.4591	26	710	3.6620	11	514	2.1401
3	52	966	5.3830	25	637	3.9246	12	563	2.1314
4	24	534	4.4944	15	576	2.6042	9	419	2.1480
Average	41.5	855.5	4.8646	24.75	676	3.6040	12.5	602.5	2.0972
SD	12.1518	248.9197	0.6610	7.4106	88.8857	0.7052	3.8730	216.0995	0.0855

Day7									
	Ctrl group			NAC group			Sham group		
Sample	8-OHdG-positive cells	Total counted cells	%	8-OHdG-positive cells	Total counted cells	%	8-OHdG-positive cells	Total counted cells	%
1	42	1089	3.8567	23	971	2.3687	6	684	0.8772
2	45	981	4.5872	58	1273	4.5562	6	461	1.3015
3	38	859	4.4237	21	780	2.6923	8	532	1.5038
4	38	824	4.6117	29	1368	2.1199	7	446	1.5695
Average	40.75	938.25	4.3698	32.75	1098	2.9343	6.75	530.75	1.3130
SD	3.4034	120.9501	0.3521	17.1731	271.2797	1.1064	0.9574	108.8344	0.3121

Day14									
	Ctrl group			NAC group			Sham group		
Sample	8-OHdG-positive cells	Total counted cells	%	8-OHdG-positive cells	Total counted cells	%	8-OHdG-positive cells	Total counted cells	%
1	42	886	4.7404	28	1015	2.7586	7	560	1.25
2	50	1109	4.5086	19	824	2.3058	7	637	1.0989
3	52	1168	4.4521	26	968	2.6860	9	592	1.5203
4	78	1029	7.5802	26	797	3.2622	21	421	4.9881
Average	55.5	1048	5.3203	24.75	901	2.7532	11	552.5	2.2143
SD	15.6098	122.1011	1.5117	3.9476	106.8176	0.3932	6.7330	93.1826	1.8574

Figure 3.

GAS

Day1									
	Ctrl group			NAC group			Sham group		
Sample	TUNEL-positive nuclei	Total counted nuclei	%	TUNEL-positive nuclei	Total counted nuclei	%	TUNEL-positive nuclei	Total counted nuclei	%
1	280	2470	11.3360	123	1876	6.5565	10	1531	0.6532
2	202	1821	11.0928	33	1551	2.1277	10	1618	0.6180
3	219	2182	10.0367	30	1894	1.5839	18	1355	1.3284
4	137	1095	12.5114	59	1302	4.5315	1	1295	0.0772
Average	209.5	1892	11.2442	61.25	1655.75	3.6999	9.75	1449.75	0.6692
SD	58.8019	593.9792	1.0158	43.1770	283.6575	2.2950	6.9462	150.3715	0.5125

Day7									
	Ctrl group			NAC group			Sham group		
Sample	TUNEL-positive nuclei	Total counted nuclei	%	TUNEL-positive nuclei	Total counted nuclei	%	TUNEL-positive nuclei	Total counted nuclei	%
1	64	3912	1.6360	16	3511	0.4557	9	1660	0.5422
2	77	4828	1.5949	5	1421	0.3519	11	1590	0.6918
3	59	3434	1.7181	58	4238	1.3686	7	1614	0.4337
4	61	4979	1.2251	43	3543	1.2137	4	1669	0.2397
Average	65.25	4288.25	1.5435	30.5	3178.25	0.8475	7.75	1633.25	0.4768
SD	8.0984	739.3179	0.2184	24.3105	1218.5733	0.5179	2.9861	37.5710	0.1903

Day14									
	Ctrl group			NAC group			Sham group		
Sample	TUNEL-positive nuclei	Total counted nuclei	%	TUNEL-positive nuclei	Total counted nuclei	%	TUNEL-positive nuclei	Total counted nuclei	%
1	64	4987	1.2833	13	2002	0.6494	5	1242	0.4026
2	44	2402	1.8318	8	1667	0.4799	12	1442	0.8322
3	33	3585	0.9205	5	2329	0.2147	5	1290	0.3876
4	42	3829	1.0969	36	3364	1.0702	4	1401	0.2855
Average	45.75	3700.75	1.2831	15.5	2340.5	0.6035	6.5	1343.75	0.4770
SD	13.0735	1060.0372	0.3946	14.0594	733.9094	0.3589	3.6968	93.4037	0.2425

SOL

Day1									
	Ctrl group			NAC group			Sham group		
Sample	TUNEL-positive nuclei	Total counted nuclei	%	TUNEL-positive nuclei	Total counted nuclei	%	TUNEL-positive nuclei	Total counted nuclei	%
1	54	1494	3.6145	21	1264	1.6614	9	1158	0.7772
2	25	1250	2.0000	6	1092	0.5495	2	1070	0.1869
3	73	1263	5.7799	3	1244	0.2412	5	1075	0.4651
4	13	970	1.3402	7	977	0.7165	2	830	0.2410
Average	41.25	1244.25	3.1836	9.25	1144.25	0.7921	4.5	1033.25	0.4175
SD	27.2809	214.4549	1.9770	8.0156	135.3917	0.6120	3.3166	141.3822	0.2683

Day7									
	Ctrl group			NAC group			Sham group		
Sample	TUNEL-positive nuclei	Total counted nuclei	%	TUNEL-positive nuclei	Total counted nuclei	%	TUNEL-positive nuclei	Total counted nuclei	%
1	9	1748	0.5149	4	1409	0.2839	4	1002	0.3992
2	19	1223	1.5536	19	1582	1.2010	6	1029	0.5831
3	14	1408	0.9943	10	1473	0.6789	4	1016	0.3937
4	19	1265	1.5020	10	1372	0.7289	3	966	0.3106
Average	15.25	1411	1.1412	10.75	1459	0.7232	4.25	1003.25	0.4216
SD	4.7871	238.2142	0.4879	6.1847	92.0036	0.3756	1.2583	27.1708	0.1150

Day14									
	Ctrl group			NAC group			Sham group		
Sample	TUNEL-positive nuclei	Total counted nuclei	%	TUNEL-positive nuclei	Total counted nuclei	%	TUNEL-positive nuclei	Total counted nuclei	%
1	15	1146	1.3089	9	1362	0.6608	1	677	0.1477
2	13	1277	1.0180	10	974	1.0267	2	848	0.2358
3	6	1277	0.4699	2	1433	0.1396	3	931	0.3222
4	13	1204	1.0797	4	1278	0.3130	3	1061	0.2828
Average	11.75	1226	0.9691	6.25	1261.75	0.5350	2.25	879.25	0.2471
SD	3.9476	63.4718	0.3556	3.8622	202.0237	0.3930	0.9574	160.8237	0.0751

Figure 4.

Day1						
	Ctrl group			NAC group		
Sample	Ischemic limb blood flow (mL/min/100g)	Contralateral control limb blood flow (mL/min/100g)	Ischemic / Contralateral ratio	Ischemic limb blood flow (mL/min/100g)	Contralateral control limb blood flow (mL/min/100g)	Ischemic / Contralateral ratio
1	17.5405	14.4692	1.2123	9.2891	8.2843	1.1213
2	12.0334	9.5706	1.2573	9.3204	8.2886	1.1245
3	11.1528	10.0675	1.1078	10.4830	8.5796	1.2218
4	11.7651	10.6138	1.1085	11.4090	10.6638	1.0699
Average	13.1229	11.1803	1.1715	10.1254	8.9541	1.1344
SD	2.9680	2.2336	0.0754	1.0203	1.1482	0.0635

Day7						
	Ctrl group			NAC group		
Sample	Ischemic limb blood flow (mL/min/100g)	Contralateral control limb blood flow (mL/min/100g)	Ischemic / Contralateral ratio	Ischemic limb blood flow (mL/min/100g)	Contralateral control limb blood flow (mL/min/100g)	Ischemic / Contralateral ratio
1	12.1250	12.1306	0.9995	10.3229	8.5704	1.2045
2	12.2005	12.4795	0.9776	13.2956	10.2951	1.2914
3	10.7008	9.4504	1.1323	10.0132	9.0961	1.1008
4	9.2504	9.1182	1.0145	11.3298	9.1267	1.2414
Average	11.0692	10.7947	1.0310	11.2404	9.2720	1.2095
SD	1.3950	1.7551	0.0692	1.4809	0.7283	0.0808

Day14						
	Ctrl group			NAC group		
Sample	Ischemic limb blood flow (mL/min/100g)	Contralateral control limb blood flow (mL/min/100g)	Ischemic / Contralateral ratio	Ischemic limb blood flow (mL/min/100g)	Contralateral control limb blood flow (mL/min/100g)	Ischemic / Contralateral ratio
1	10.5171	10.4406	1.0073	10.5511	9.0634	1.1641
2	11.1238	10.2311	1.0873	10.7937	9.9563	1.0841
3	10.5341	10.4629	1.0068	10.1658	10.5132	0.9670
4	11.4101	10.9745	1.0397	10.1721	10.1157	1.0056
Average	10.8963	10.5273	1.0353	10.4207	9.9122	1.0552
SD	0.4437	0.3159	0.0379	0.3071	0.6124	0.0875

Figure 5.

GAS

Day1									
	Ctrl group			NAC group			Sham group		
Sample	Collagen-positive area (μm²)	Total tissue area (μm²)	%	Collagen-positive area (μm²)	Total tissue area (μm²)	%	Collagen-positive area (μm²)	Total tissue area (μm²)	%
1	17859.86	704197.98	2.5362	11566.27	596273.63	1.9398	8503.86	871721.76	0.9755
2	38693.3	782874.96	4.9425	20442.27	879733.3	2.3237	4971.58	809885.6	0.6139
3	20859.19	684761.53	3.0462	18352.67	886170.64	2.0710	7038.28	777338.27	0.9054
4	31673.64	790773.96	4.0054	17693.68	736892.69	2.4011	6207.55	777705.68	0.7982
Average	27271.4975	740652.1075	3.6326	17013.7225	774767.5650	2.1839	6680.3175	809162.8275	0.8233
SD	9652.8586	53998.8563	1.0647	3815.9664	137505.2019	0.2153	1482.8730	44409.0720	0.1575

Day7									
	Ctrl group			NAC group			Sham group		
Sample	Collagen-positive area (μm²)	Total tissue area (μm²)	%	Collagen-positive area (μm²)	Total tissue area (μm²)	%	Collagen-positive area (μm²)	Total tissue area (μm²)	%
1	71613.91	751602.60	9.5282	53603.19	789649.76	6.7882	7520.83	780210.06	0.9639
2	77872.35	725377.89	10.7354	14032.86	784240.25	1.7894	11862.09	805573.02	1.4725
3	66230.30	818904.11	8.0877	51473.70	857095.54	6.0056	8469.13	829509.61	1.0210
4	53160.95	772244.78	6.8840	32387.42	729652.26	4.4387	10239.26	795426.60	1.2873
Average	67219.3775	767032.3450	8.8088	37874.2925	790159.4525	4.7555	9522.8275	802679.8225	1.1862
SD	10510.5566	39543.3102	1.6787	18537.0060	52207.4608	2.2055	1923.8587	20701.9126	0.2373

Day14									
	Ctrl group			NAC group			Sham group		
Sample	Collagen-positive area (μm²)	Total tissue area (μm²)	%	Collagen-positive area (μm²)	Total tissue area (μm²)	%	Collagen-positive area (μm²)	Total tissue area (μm²)	%
1	119194.44	781974.28	15.2428	32294.25	735050.05	4.3935	4595.44	727005.03	0.6321
2	95847.54	887061.78	10.8051	61795.30	799090.56	7.7332	3673.39	919593.94	0.3995
3	82868.08	781137.24	10.6086	37890.84	851356.56	4.4506	11198.18	693273.87	1.6153
4	86370.02	852954.20	10.1260	61256.62	793002.22	7.7246	12265.43	785427.21	1.5616
Average	96070.0200	825781.8750	11.6956	48309.2525	794624.8475	6.0755	7933.1100	781325.0125	1.0521
SD	16362.2484	52933.3254	2.3819	15432.9888	47575.2709	1.9094	4423.9852	99730.9523	0.6269

SOL

Day1									
	Ctrl group			NAC group			Sham group		
Sample	Collagen-positive area (μm²)	Total tissue area (μm²)	%	Collagen-positive area (μm²)	Total tissue area (μm²)	%	Collagen-positive area (μm²)	Total tissue area (μm²)	%
1	8934.02	370764.74	2.4096	6797.84	384382.74	1.7685	4739.47	356150.92	1.3307
2	10202.76	377358.43	2.7037	8444.60	401095.82	2.1054	2557.06	402579.96	0.6352
3	9652.11	346335.2	2.7869	8833.98	443462.72	1.9920	3633.43	373880.50	0.9718
4	8809.66	391963.13	2.2476	4858.14	349986.68	1.3881	5010.43	376927.02	1.3293
Average	9399.6375	371605.3750	2.5370	7233.6400	394731.9900	1.8135	3985.0975	377384.6000	1.0668
SD	651.5677	19033.5164	0.2518	1812.9565	38834.8302	0.3163	1122.9939	19132.5502	0.3336

Day7									
	Ctrl group			NAC group			Sham group		
Sample	Collagen-positive area (μm²)	Total tissue area (μm²)	%	Collagen-positive area (μm²)	Total tissue area (μm²)	%	Collagen-positive area (μm²)	Total tissue area (μm²)	%
1	17199.83	363763.36	4.7283	10146.45	405749.81	2.5007	9962.25	364550.25	2.7328
2	23386.72	370180.47	6.3177	23038.47	379801.34	6.0659	10437.50	412731.71	2.5289
3	23408.34	411725.82	5.6854	13207.54	398089.55	3.3177	5302.20	360104.54	1.4724
4	21062.98	364602.53	5.7770	14098.00	343597.21	4.1031	5792.30	371434.56	1.5594
Average	21264.4675	377568.0450	5.6271	15122.6150	381809.4775	3.9968	7873.5625	377205.2650	2.0734
SD	2924.7234	22949.2466	0.6609	5541.9710	27703.1303	1.5267	2700.6141	24138.5827	0.6500

Day14									
	Ctrl group			NAC group			Sham group		
Sample	Collagen-positive area (μm²)	Total tissue area (μm²)	%	Collagen-positive area (μm²)	Total tissue area (μm²)	%	Collagen-positive area (μm²)	Total tissue area (μm²)	%
1	35247.95	319583.84	11.0293	11289.32	358525.70	3.1488	5339.56	309411.28	1.7257
2	25266.97	438396.37	5.7635	3970.75	362973.83	1.0939	5476.82	405128.75	1.3519
3	8532.25	257944.82	3.3078	9387.73	444572.08	2.1116	4274.33	347538.93	1.2299
4	17683.42	333907.36	5.2959	15853.70	428699.82	3.6981	7545.05	426728.71	1.7681
Average	21682.6475	337458.0975	6.3491	10125.3750	398692.8575	2.5131	5658.9400	372201.9175	1.5189
SD	11340.0702	74928.8395	3.2968	4919.0787	44326.7050	1.1523	1367.4462	53567.0126	0.2685

Figure 6

A.

	Fluorescene Intensity (a.u.)		
Sample	Control group<br>H_2O_2(-) NAC (-)	H_2O_2 group<br>H_2O_2(+) NAC (-)	NAC group<br>H_2O_2(+) NAC (+)
1	15509	17112	15272
2	16777	16713	15947
3	16618	17368	15236
4	15953	17143	15464
5	15895	17517	15949
6	16441	18312	15773
Average	16198.8333	17360.8333	15606.8333
SD	489.3344	540.0398	325.7099

B.

	GSH levels (μM)		
Sample	Control group<br>H_2O_2(-) NAC (-)	H_2O_2 group<br>H_2O_2(+) NAC (-)	NAC group<br>H_2O_2(+) NAC (+)
1	0.8352	0.3234	1.2600
2	1.0379	0.5986	1.3566
3	1.0717	0.6903	1.3228
Average	0.9816	0.5375	1.3131
SD	0.1279	0.1909	0.0490

C.

	Gapdh	*Bcl2*						
Sample	CT	CT	ΔCT	Average	ΔΔCT	Fold change	Average	SD
Control group_1	28.58	29.54	0.96	1.1083	-0.148	1.108288387	1.003962	0.097651
Control group_2	28.94	30.18	1.24		0.132	0.91277636		
Control group_3	29.1	30.38	1.28		0.172	0.887816443		
Control group_4	29.35	30.51	1.16		0.052	0.96482108		
Control group_5	29.14	30.2	1.06		-0.048	1.034069629		
Control group_6	28.87	29.82	0.95		-0.158	1.115997143		
H_2O_2 group_1	28.6	29.39	0.79	0.6067	-0.318	1.246889254	1.437734	0.271946
H_2O_2 group_2	28.76	29.56	0.8		-0.308	1.23827636		
H_2O_2 group_3	28.36	28.73	0.37		-0.738	1.66824749		
H_2O_2 group_4	28.69	29.01	0.32		-0.788	1.727078111		
H_2O_2 group_5	28.31	28.7	0.39		-0.718	1.645280234		
H_2O_2 group_6	27.89	28.86	0.97		-0.138	1.10063288		
NAC group_1	27.38	27.43	0.05	-0.4667	-1.058	2.082524305	3.065884	0.797201
NAC group_2	28.14	27.71	-0.43		-1.538	2.904587585		
NAC group_3	28.7	27.71	-0.99		-2.098	4.282144065		
NAC group_4	28.8	28.13	-0.67		-1.778	3.430296622		
NAC group_5	28.82	28.19	-0.63		-1.738	3.33649498		
NAC group_6	28.34	28.21	-0.13		-1.238	2.359258226		

	Gapdh	*BAX*						
Sample	CT	CT	ΔCT	Average	ΔΔCT	Fold change	Average	SD
Control group_1	27.64	26.61	-1.03	-2.0250	0.995	0.501735874	1.054210	0.334849
Control group_2	28.39	26.56	-1.83		0.195	0.873572896		
Control group_3	28.63	26.49	-2.14		-0.115	1.082975046		
Control group_4	29.15	26.56	-2.59		-0.565	1.479387509		
Control group_5	28.77	26.45	-2.32		-0.295	1.226884977		
Control group_6	28.89	26.65	-2.24		-0.215	1.160703914		
H_2O_2 group_1	28.12	25.13	-2.99	-3.3733	-0.965	1.952063522	2.618780	0.650461
H_2O_2 group_2	27.93	24.44	-3.49		-1.465	2.760634707		
H_2O_2 group_3	27.99	24.38	-3.61		-1.585	3.000077979		
H_2O_2 group_4	28.37	24.56	-3.81		-1.785	3.446184639		
H_2O_2 group_5	28.17	24.65	-3.52		-1.495	2.818641510		
H_2O_2 group_6	27.76	24.94	-2.82		-0.795	1.735077374		
NAC group_1	27.18	24.38	-2.8	-3.0033	-0.775	1.711190051	1.979718	0.214642
NAC group_2	27.62	24.38	-3.24		-1.215	2.321407829		
NAC group_3	27.95	24.89	-3.06		-1.035	2.049113646		
NAC group_4	28.12	25.05	-3.07		-1.045	2.063366359		
NAC group_5	28.30	25.34	-2.96		-0.935	1.911890635		
NAC group_6	28.22	25.33	-2.89		-0.865	1.821339667		

	Gapdh	*Casp3*						
Sample	CT	CT	ΔCT	Average	ΔΔCT	Fold change	Average	SD
Control group_1	28.15	28.46	0.31	0.2150	0.095	0.936272247	1.065952	0.358192
Control group_2	28.51	28.17	-0.34		-0.555	1.469168633		
Control group_3	28.58	28.5	-0.08		-0.295	1.226884977		
Control group_4	28.48	28.32	-0.16		-0.375	1.296839555		
Control group_5	27.81	28.00	0.19		-0.025	1.017479692		
Control group_6	27.72	29.09	1.37		1.155	0.449066186		
H_2O_2 group_1	28.35	27	-1.35	-1.6150	-1.565	2.958775018	3.576236	0.419800
H_2O_2 group_2	28.24	26.39	-1.85		-2.065	4.184339759		
H_2O_2 group_3	28.28	26.58	-1.7		-1.915	3.771138144		
H_2O_2 group_4	28.23	26.58	-1.65		-1.865	3.642679334		
H_2O_2 group_5	28.05	26.55	-1.5		-1.715	3.282966435		
H_2O_2 group_6	28.21	26.57	-1.64		-1.855	3.61751751		
NAC group_1	27.46	26.45	-1.01	-1.1567	-1.225	2.337554497	2.630898	0.511207
NAC group_2	27.93	26.81	-1.12		-1.335	2.522754818		
NAC group_3	28.36	26.95	-1.41		-1.625	3.084421651		
NAC group_4	28.65	27.44	-1.21		-1.425	2.685145006		
NAC group_5	28.72	27.22	-1.5		-1.715	3.282966435		
NAC group_6	28.08	27.39	-0.69		-0.905	1.872544495		

Figure 7

A.

	Gapdh	*Col1a1*						
Sample	CT	CT	ΔCT	Average	ΔΔCT	Fold change	Average	SD
Control group_1	28.6	25.47	-3.13	-3.3717	0.242	0.845767679	1.005070	0.110150
Control group_2	28.66	25.31	-3.35		0.022	0.985094022		
Control group_3	28.66	25.12	-3.54		-0.168	1.123759517		
Control group_4	28.78	25.22	-3.56		-0.188	1.139446615		
Control group_5	28.33	25.01	-3.32		0.052	0.96482108		
Control group_6	28.71	25.38	-3.33		0.042	0.971531941		
H_2O_2 group_1	28.37	23.89	-4.48	-4.7050	-1.108	2.155964366	2.567072	0.541996
H_2O_2 group_2	28.62	23.96	-4.66		-1.288	2.442457288		
H_2O_2 group_3	36.76	32.47	-4.29		-0.918	1.889930698		
H_2O_2 group_4	28.13	23.15	-4.98		-1.608	3.048994047		
H_2O_2 group_5	28.52	23.41	-5.11		-1.738	3.33649498		
H_2O_2 group_6	28.55	23.84	-4.71		-1.338	2.528590358		
NAC (4μM) group_1	27.93	26.63	-1.3	-2.0217	2.072	0.237884526	0.406867	0.112528
NAC (4μM) group_2	28.83	26.59	-2.24		1.132	0.456388180		
NAC (4μM) group_3	28.36	26.64	-1.72		1.652	0.318272262		
NAC (4μM) group_4	28.44	26.01	-2.43		0.942	0.520631076		
NAC (4μM) group_5	28.19	26.16	-2.03		1.342	0.394564574		
NAC (4μM) group_6	28.41	26	-2.41		0.962	0.513463394		
NAC (20μM) group_1	28.65	26.61	-2.04	-2.3683	1.332	0.397308987	0.513999	0.131142
NAC (20μM) group_2	28.8	26.42	-2.38		0.992	0.502896471		
NAC (20μM) group_3	28.74	26.13	-2.61		0.762	0.589814556		
NAC (20μM) group_4	28.82	26.02	-2.8		0.572	0.672839045		
NAC (20μM) group_5	28.79	26.17	-2.62		0.752	0.593917041		
NAC (20μM) group_6	27.95	26.19	-1.76		1.612	0.327220113		
NAC (100μM) group_1	27.82	26.29	-1.53	-1.7917	1.842	0.278999286	0.338096	0.053691
NAC (100μM) group_2	28.36	26.59	-1.77		1.602	0.329496109		
NAC (100μM) group_3	28.43	26.47	-1.96		1.412	0.375877206		
NAC (100μM) group_4	28.46	26.38	-2.08		1.292	0.408478863		
NAC (100μM) group_5	28.28	26.38	-1.9		1.472	0.360565517		
NAC (100μM) group_6	27.99	26.48	-1.51		1.862	0.27515822		
NAC (500μM) group_1	28.06	26.38	-1.68	-1.5767	1.692	0.30956909	0.294139	0.060604
NAC (500μM) group_2	28.54	26.92	-1.62		1.752	0.296958521		
NAC (500μM) group_3	28.87	27.4	-1.47		1.902	0.267634004		
NAC (500μM) group_4	28.92	27.07	-1.85		1.522	0.348283331		
NAC (500μM) group_5	29.02	27.15	-1.87		1.502	0.353145186		
NAC (500μM) group_6	27.92	26.95	-0.97		2.402	0.189245819		

	Gapdh	*Col3*						
Sample	CT	CT	ΔCT	Average	ΔΔCT	Fold change	Average	SD
Control group_1	28.61	22.82	-5.79	-5.7317	-0.058	1.041262153	1.004456	0.099785
Control group_2	28.45	22.78	-5.67		0.062	0.958156574		
Control group_3	28.44	22.58	-5.86		-0.128	1.093030254		
Control group_4	28.29	22.47	-5.82		-0.088	1.063141284		
Control group_5	28.11	22.31	-5.8		-0.068	1.048504704		
Control group_6	27.75	22.3	-5.45		0.282	0.822640117		
H_2O_2 group_1	28.12	21.38	-6.74	-7.0683	-1.008	2.011585882	2.588690	0.646408
H_2O_2 group_2	28.41	21.36	-7.05		-1.318	2.493778507		
H_2O_2 group_3	28.12	20.52	-7.6		-1.868	3.651105441		
H_2O_2 group_4	28.23	20.91	-7.32		-1.588	3.007017644		
H_2O_2 group_5	28.31	21.3	-7.01		-1.278	2.425586003		
H_2O_2 group_6	27.83	21.14	-6.69		-0.958	1.943063882		
NAC (4μM) group_1	28.07	21.98	-6.09	-6.9100	-0.358	1.281944082	2.336257	0.570317
NAC (4μM) group_2	28.13	21.26	-6.87		-1.138	2.201265760		
NAC (4μM) group_3	28.45	21.19	-7.26		-1.528	2.884524133		
NAC (4μM) group_4	28.05	20.86	-7.19		-1.458	2.747907295		
NAC (4μM) group_5	28.38	21.35	-7.03		-1.298	2.459445922		
NAC (4μM) group_6	28.33	21.31	-7.02		-1.288	2.442457288		
NAC (20μM) group_1	27.7	20.92	-6.78	-6.9267	-1.048	2.068139259	2.368603	0.622156
NAC (20μM) group_2	28.58	21.68	-6.9		-1.168	2.247519033		
NAC (20μM) group_3	28.69	21.42	-7.27		-1.538	2.904587585		
NAC (20μM) group_4	28.82	21.49	-7.33		-1.598	3.027933106		
NAC (20μM) group_5	28.47	21.35	-7.12		-1.388	2.617760904		
NAC (20μM) group_6	27.25	21.09	-6.16		-0.428	1.345678090		
NAC (100μM) group_1	27.42	21.18	-6.24	-6.4750	-0.508	1.422406018	1.712073	0.400279
NAC (100μM) group_2	28.34	21.66	-6.68		-0.948	1.929642160		
NAC (100μM) group_3	28.64	21.69	-6.95		-1.218	2.326777621		
NAC (100μM) group_4	28.06	21.43	-6.63		-0.898	1.863911464		
NAC (100μM) group_5	28.11	21.81	-6.3		-0.568	1.482809572		
NAC (100μM) group_6	27.76	21.71	-6.05		-0.318	1.246889254		
NAC (500μM) group_1	27.96	21.71	-6.25	-6.2083	-0.518	1.432299634	1.399989	0.172355
NAC (500μM) group_2	28.36	22.24	-6.12		-0.388	1.308880452		
NAC (500μM) group_3	28.73	22.56	-6.17		-0.438	1.355038022		
NAC (500μM) group_4	28.65	22.15	-6.5		-0.768	1.703300916		
NAC (500μM) group_5	28.75	22.52	-6.23		-0.498	1.412580742		
NAC (500μM) group_6	27.89	21.91	-5.98		-0.248	1.187834082		

B.

	Gapdh	*Tgfb1*						
Sample	CT	CT	ΔCT	Average	ΔΔCT	Fold change	Average	SD
Control group_1	27.76	26.08	-1.68	-1.6383	-0.042	1.029302237	1.002988	0.084523
Control group_2	26.89	25.23	-1.66		-0.022	1.015131529		
Control group_3	26.96	25.28	-1.68		-0.042	1.029302237		
Control group_4	26.72	25.26	-1.46		0.178	0.883723324		
Control group_5	27.07	25.26	-1.81		-0.172	1.126358954		
Control group_6	27.4	25.86	-1.54		0.098	0.934111496		
H_2O_2 group_1	27.75	25.22	-2.53	-2.8367	-0.892	1.855318234	2.331642	0.458288
H_2O_2 group_2	27.99	25.27	-2.72		-1.082	2.116479723		
H_2O_2 group_3	28.9	25.73	-3.17		-1.532	2.891196504		
H_2O_2 group_4	28.3	25.41	-2.89		-1.252	2.38116347		
H_2O_2 group_5	28.08	25.52	-2.56		-0.922	1.894302414		
H_2O_2 group_6	28.6	25.45	-3.15		-1.512	2.851392547		
NAC group_1	28.19	25.72	-2.47	-2.5217	-0.832	1.779740212	1.868274	0.331483
NAC group_2	27.59	25.37	-2.22		-0.582	1.496577164		
NAC group_3	28.06	25.19	-2.87		-1.232	2.348381288		
NAC group_4	27.95	25.18	-2.77		-1.132	2.191117219		
NAC group_5	27.86	25.52	-2.34		-0.702	1.626382579		
NAC group_6	27.85	25.39	-2.46		-0.822	1.767446648		

	Gapdh	*Tgfb2*						
Sample	CT	CT	ΔCT	Average	ΔΔCT	Fold change	Average	SD
Control group_1	27.31	27.9	0.59	0.6283	-0.038	1.026926789	1.004062	0.1002052
Control group_2	27.04	27.75	0.71		0.082	0.944965349		
Control group_3	27.33	27.74	0.41		-0.218	1.16338881		
Control group_4	27.12	27.91	0.79		0.162	0.893991694		
Control group_5	28.13	28.67	0.54		-0.088	1.063141284		
Control group_6	28.06	28.79	0.73		0.102	0.931955732		
H_2O_2 group_1	28.18	28.19	0.01	-0.241	-0.618	1.535100739	1.841687	0.2475868
H_2O_2 group_2	27.96	27.61	-0.35		-0.978	1.970188043		
H_2O_2 group_3	27.73	27.7	-0.03		-0.658	1.578258295		
H_2O_2 group_4	28.62	28.38	-0.24		-0.868	1.825552721		
H_2O_2 group_5	28.84	28.39	-0.45		-1.078	2.111595261		
H_2O_2 group_6	28.02	27.87	-0.15		-0.778	1.715148311		
H_2O_2 group_7	28.6	28.12	-0.48		-1.108	2.155964366		
NAC group_1	27.62	27.82	0.2	-0.178	-0.428	1.34567809	1.764435	0.2437878
NAC group_2	28.16	27.97	-0.19		-0.818	1.763367693		
NAC group_3	28.22	27.91	-0.31		-0.938	1.916313148		
NAC group_4	28.22	27.85	-0.37		-0.998	1.997690843		
NAC group_5	28.29	27.97	-0.32		-0.948	1.92964216		
NAC group_6	28	27.92	-0.08		-0.708	1.633915453		

	Gapdh	*Fgf2*						
Sample	CT	CT	ΔCT	Average	ΔΔCT	Fold change	Average	SD
Control group_1	29.56	32.12	2.56	2.7383	-0.178	1.131575882	1.022474	0.216009
Control group_2	29.6	32.53	2.93		0.192	0.875593611		
Control group_3	29.83	32.34	2.51		-0.228	1.171480819		
Control group_4	29.71	32.23	2.52		-0.218	1.163388810		
Control group_5	29.79	32.33	2.54		-0.198	1.147372093		
Control group_6	28.9	32.27	3.37		0.632	0.645430354		
H_2O_2 group_1	28.43	30.23	1.8	1.7700	-0.938	1.916313148	1.971990	0.282492
H_2O_2 group_2	28.43	30.21	1.78		-0.958	1.943063882		
H_2O_2 group_3	28.44	29.85	1.41		-1.328	2.511124108		
H_2O_2 group_4	28.51	30.31	1.8		-0.938	1.916313148		
H_2O_2 group_5	28.59	30.59	2		-0.738	1.668247490		
H_2O_2 group_6	28.38	30.21	1.83		-0.908	1.876875993		
NAC group_1	28.52	30.75	2.23	1.9683	-0.508	1.422406018	1.739217	0.357959
NAC group_2	28.81	30.52	1.71		-1.028	2.039666575		
NAC group_3	28.58	30.26	1.68		-1.058	2.082524305		
NAC group_4	28.01	30.5	2.49		-0.248	1.187834082		
NAC group_5	28.87	30.69	1.82		-0.918	1.889930698		
NAC group_6	28.82	30.7	1.88		-0.858	1.812942707		

	Gapdh	*Ccn2*						
Sample	CT	CT	ΔCT	Average	ΔΔCT	Fold change	Average	SD
Control group_1	29.21	24.32	-4.89	-4.8550	-0.035	1.024556823	1.026457	0.231279
Control group_2	28.77	23.72	-5.05		-0.195	1.144724161		
Control group_3	28.6	23.45	-5.15		-0.295	1.226884977		
Control group_4	29.21	24.09	-5.12		-0.265	1.201636050		
Control group_5	28.94	24.15	-4.79		0.065	0.955945318		
Control group_6	27.94	23.81	-4.13		0.725	0.604997045		
H_2O_2 group_1	27.57	22.89	-4.68	-5.6150	0.175	0.885767519	1.756603	0.439536
H_2O_2 group_2	27.95	22.13	-5.82		-0.965	1.952063522		
H_2O_2 group_3	27.64	21.93	-5.71		-0.855	1.808758755		
H_2O_2 group_4	27.97	22.07	-5.9		-1.045	2.063366359		
H_2O_2 group_5	28.1	22.23	-5.87		-1.015	2.020902893		
H_2O_2 group_6	28.14	22.43	-5.71		-0.855	1.808758755		
NAC group_1	27.25	23.06	-4.19	-4.8450	0.665	0.630688704	1.019246	0.238562
NAC group_2	28.2	23.38	-4.82		0.035	0.976031761		
NAC group_3	28.33	23.23	-5.1		-0.245	1.185092771		
NAC group_4	28.48	23.47	-5.01		-0.155	1.113421618		
NAC group_5	28.68	23.44	-5.24		-0.385	1.305859787		
NAC group_6	27.66	22.95	-4.71		0.145	0.904379378		

C.

p-ERK

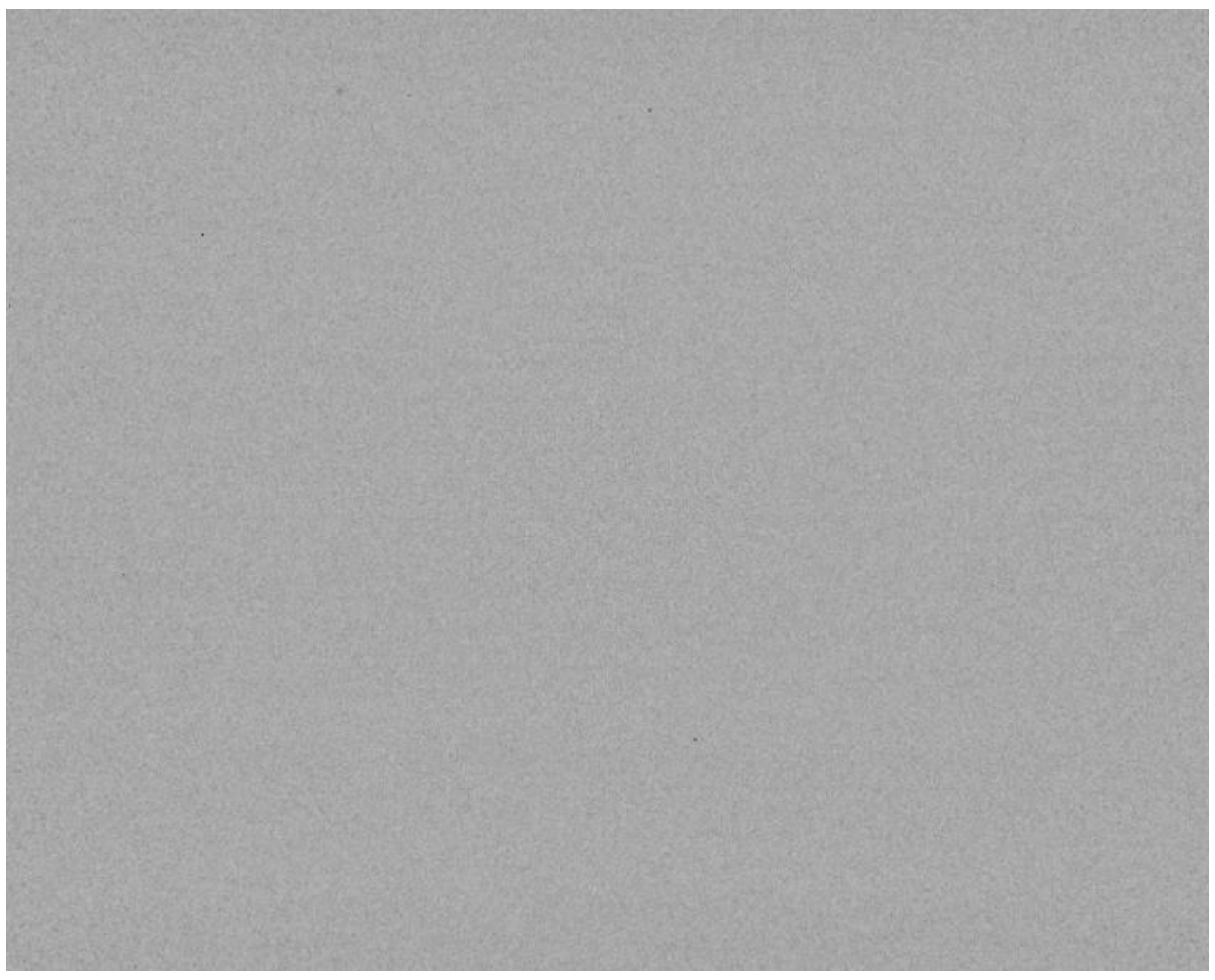

ERK

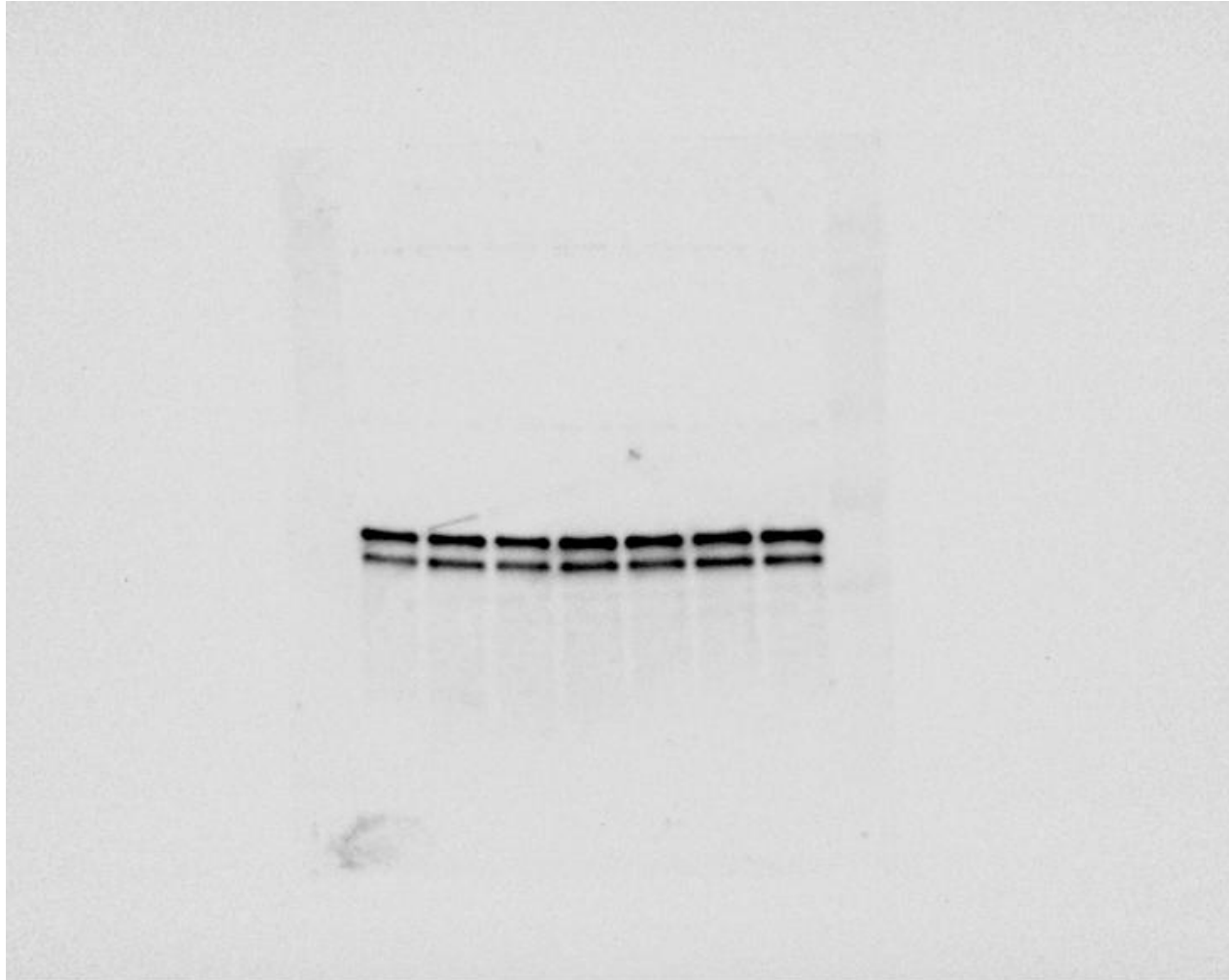

p-p38

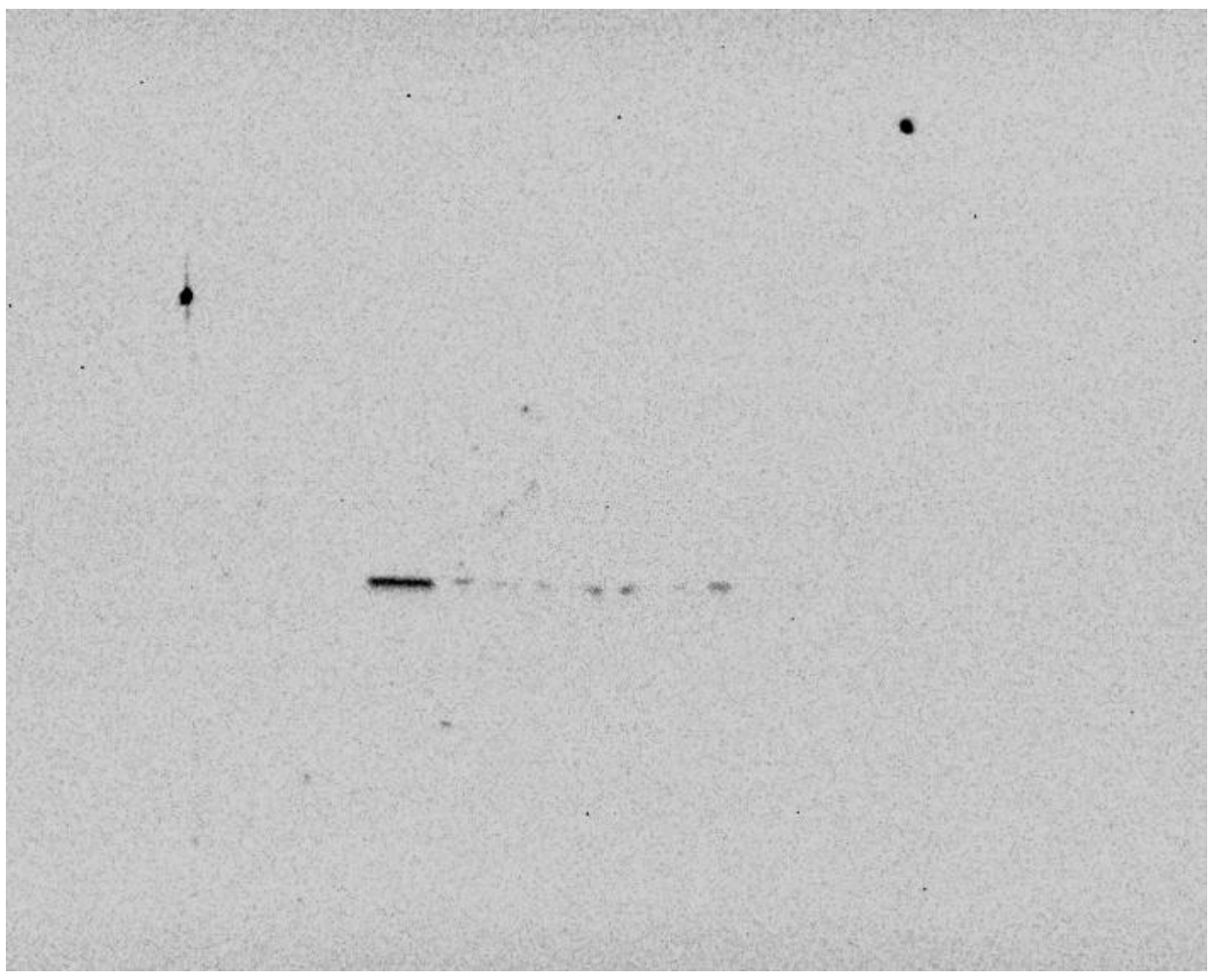

p38

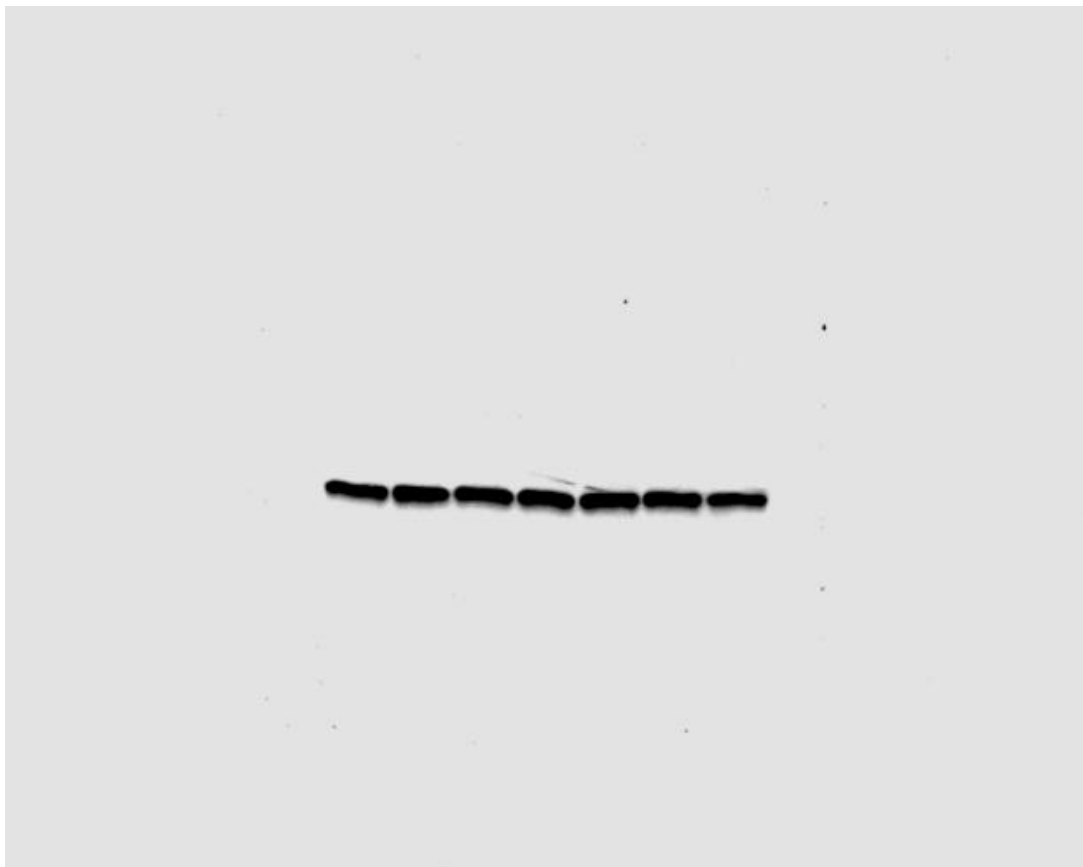

p-JNK

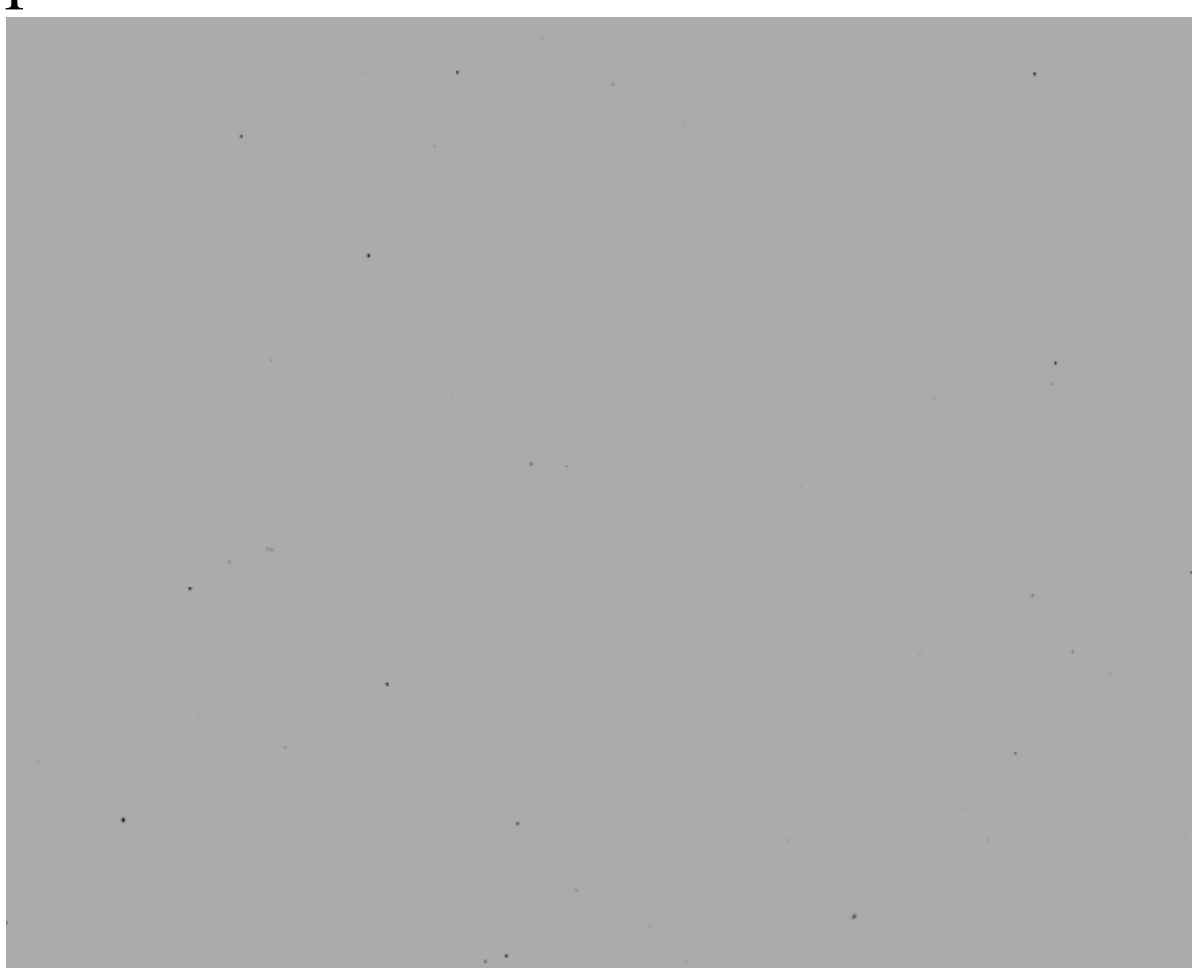

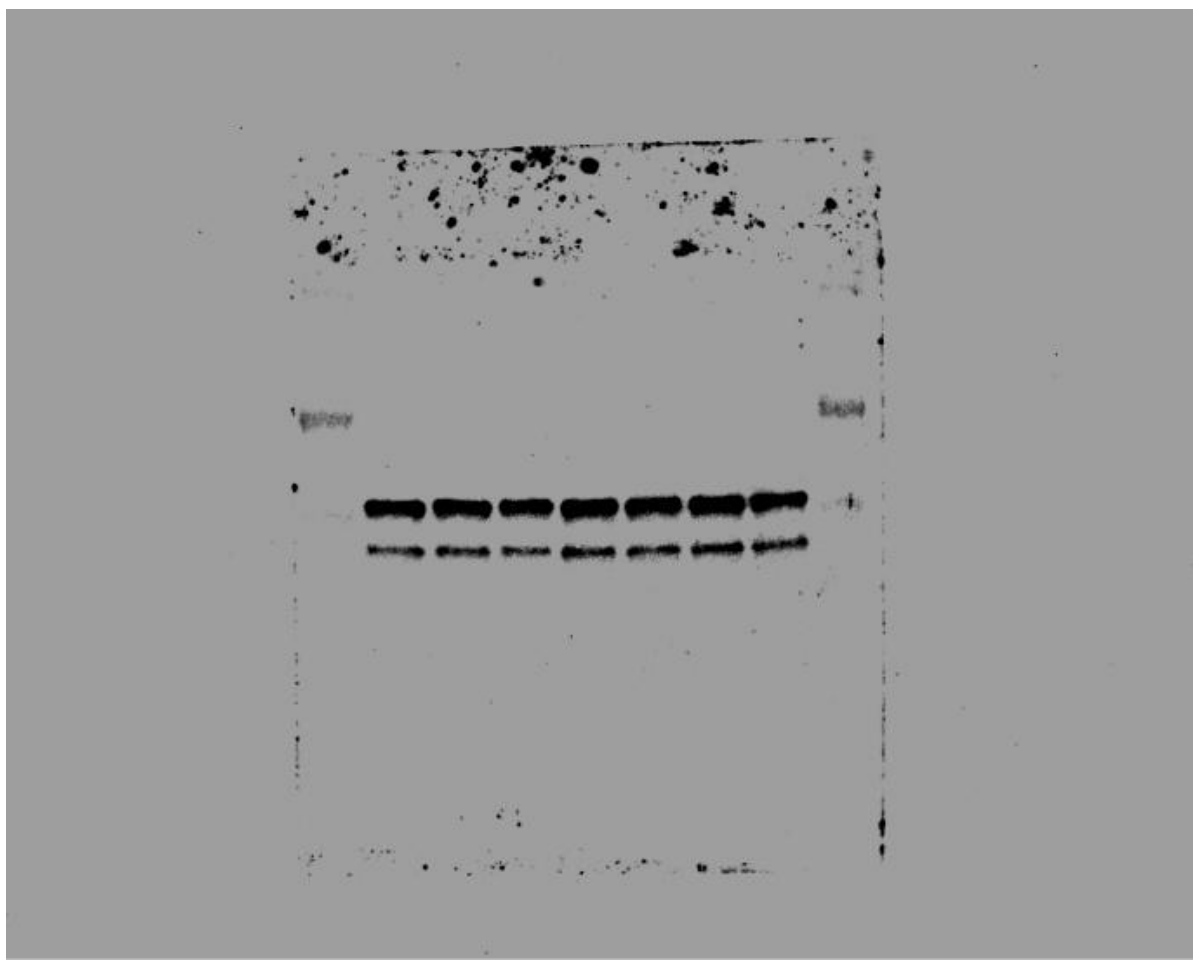

D.

	Gapdh	*Col1a1*						
Sample	CT	CT	ΔCT	Average	ΔΔCT	Fold change	Average	SD
vehicle_1	29.42	22.4	-7.02	-7.1117	0.092	0.938437997	1.002767	0.082056
vehicle_2	29.89	22.68	-7.21		-0.098	1.070536016		
vehicle_3	29.83	22.67	-7.16		-0.048	1.034069629		
vehicle_4	29.93	22.66	-7.27		-0.158	1.115997143		
vehicle_5	29.74	22.75	-6.99		0.122	0.919125222		
vehicle_6	29.73	22.71	-7.02		0.092	0.938437997		
SB203580_1	29.11	22.46	-6.65	-6.6267	0.462	0.726146896	0.727220	0.135937
SB203580_2	29.48	22.67	-6.81		0.302	0.811314587		
SB203580_3	29.63	22.82	-6.81		0.302	0.811314587		
SB203580_4	29.27	22.42	-6.85		0.262	0.834123745		
SB203580_5	29.38	22.76	-6.62		0.492	0.711203009		
SB203580_6	28.6	22.58	-6.02		1.092	0.469218998		
SP600125_1	29.85	22.	-7.25	-7.065	-0.138	1.100632880	0.983796	0.183249
SP600125_2	29.72	22.97	-6.75		0.362	0.778264973		
SP600125_3	30.37	23.12	-7.25		-0.138	1.100632880		
SP600125_4	30.25	23.04	-7.21		-0.098	1.070536016		
SP600125_5	30.23	22.94	-7.29		-0.178	1.131575882		
SP600125_6	29.82	23.18	-6.64		0.472	0.721131033		
U0126_1	29.4	22.04	-7.36	-7.1217	-0.248	1.187834082	1.026909	0.212075
U0126_2	29.8	22.71	-7.09		0.022	0.985094022		
U0126_3	30.18	22.75	-7.43		-0.318	1.246889254		
U0126_4	30.15	22.82	-7.33		-0.218	1.163388810		
U0126_5	29.74	22.81	-6.93		0.182	0.881683847		
U0126_6	29.45	22.86	-6.59		0.522	0.696566663		
NC_1	N/A	N/A						
NC_2	N/A	N/A						
NC _3	N/A	N/A						
NC _4	N/A	N/A						
NC _5	N/A	N/A						
NC _6	N/A	N/A						
